# The Effect of Iron Limitation on the Transcriptome and Proteome of *Pseudomonas fluorescens* Pf-5

**DOI:** 10.1371/journal.pone.0039139

**Published:** 2012-06-18

**Authors:** Chee Kent Lim, Karl A. Hassan, Sasha G. Tetu, Joyce E. Loper, Ian T. Paulsen

**Affiliations:** 1 Department of Chemistry and Biomolecular Sciences, Macquarie University, Sydney, Australia; 2 USDA-ARS Horticultural Crops Research Laboratory and Department of Botany and Plant Pathology, Oregon State University, Corvallis, Oregon, United States of America; Laurentian University, Canada

## Abstract

One of the most important micronutrients for bacterial growth is iron, whose bioavailability in soil is limited. Consequently, rhizospheric bacteria such as *Pseudomonas fluorescens* employ a range of mechanisms to acquire or compete for iron. We investigated the transcriptomic and proteomic effects of iron limitation on *P. fluorescens* Pf-5 by employing microarray and iTRAQ techniques, respectively. Analysis of this data revealed that genes encoding functions related to iron homeostasis, including pyoverdine and enantio-pyochelin biosynthesis, a number of TonB-dependent receptor systems, as well as some inner-membrane transporters, were significantly up-regulated in response to iron limitation. Transcription of a ribosomal protein L36-encoding gene was also highly up-regulated during iron limitation. Certain genes or proteins involved in biosynthesis of secondary metabolites such as 2,4-diacetylphloroglucinol (DAPG), orfamide A and pyrrolnitrin, as well as a chitinase, were over-expressed under iron-limited conditions. In contrast, we observed that expression of genes involved in hydrogen cyanide production and flagellar biosynthesis were down-regulated in an iron-depleted culture medium. Phenotypic tests revealed that Pf-5 had reduced swarming motility on semi-solid agar in response to iron limitation. Comparison of the transcriptomic data with the proteomic data suggested that iron acquisition is regulated at both the transcriptional and post-transcriptional levels.

## Introduction

*Pseudomonas fluorescens* Pf-5 is a soil bacterium that was first described for its capacity to protect cotton seedlings from infection by *Rhizoctonia solani* and *Pythium ultimum* [1], [2]. Subsequently, the potential of Pf-5 to control other plant pathogenic fungi and bacteria was demonstrated [3], [4]. The biocontrol activities of Pf-5 can be attributed largely to its secretion of a broad spectrum of bioactive secondary metabolites [5], and approximately 6% of the 7.1 Mbp genome of Pf-5 is dedicated to secondary metabolite production [6]. The secondary metabolites produced by Pf-5 that have been demonstrated to be antagonistic to plant pathogens are pyrrolnitrin, pyoluteorin, hydrogen cyanide, rhizoxin analogs, and 2,4-diacetylphloroglucinol (DAPG) [1], [2], [7], [8], [9]. In addition, Pf-5 produces orfamide A, a recently identified biosurfactant compound which was shown to have anti-microbial activity [10].

One of the most important micronutrients for bacterial growth is iron, which is an essential cofactor in a number of important enzymes involved in energy metabolism. Iron is abundant in soil but, under aerobic conditions, it exists primarily in the insoluble ferric oxide form [11], which is not available for microbial growth. Due to the scarcity of available iron in many microbial habitats as well as the toxicity of free iron at elevated concentrations, bacteria employ a variety of mechanisms to regulate intracellular iron concentrations [12]. A mechanism of particular importance is the production and secretion of siderophores, which are small molecules that are secreted from the cell and sequester iron from the environment through high-affinity interactions [12], [13]. Iron-complexed siderophores can then be taken up by the bacteria via siderophore specific cell-surface receptors and transport systems [14]. The production and secretion of siderophores is thought to confer a competitive advantage on the producing organism, since siderophore-bound iron is not available to surrounding organisms lacking specific uptake systems for the ferric-siderophore complexes. The production of siderophores by biological control bacteria in the rhizosphere may also suppress plant disease by sequestering iron in a form that is not available to pathogens, resulting in their iron starvation [15].

*P. fluorescens* Pf-5 produces and secretes two siderophores, a pyoverdine and enantio-pyochelin [16]. The capacity to produce these compounds is encoded in distinct gene clusters in the genome of Pf-5 that contain genes for siderophore biosynthesis, regulation, and membrane transport, including exporting the unloaded siderophores and importing iron-siderophore complexes [6], [16], [17]. In addition, Pf-5 encodes a number of TonB-dependent receptors that take up siderophores produced by other microorganisms [17], putative haem uptake membrane transporters, mechanisms for direct uptake of ferrous iron, and iron storage proteins such as bacterioferritins. Pf-5 can also utilize iron complexes of plant-produced compounds such as iron-phytosiderophore and iron-citrate [18]. As in other gamma-proteobacteria, iron acquisition by *Pseudomonas* spp. is under the control of the ferric uptake regulator (Fur) [19], [20]. Fur forms a homodimer complex with iron(II) and acts as a transcriptional repressor by binding to specific DNA sequences (Fur boxes) in the operator regions of target genes [21]. Transcriptional repression is relieved under low iron conditions, when iron(II) dissociates from the Fur complex and affinity for the Fur box is reduced. Fur is a global regulator of iron uptake, thus transcriptional control occurs both directly and also indirectly via the expression of regulatory RNAs, sigma factors, and other transcriptional regulators [19], [22], [23], [24], [25], [26].

Studies investigating the effect of iron limitation on global gene expression in the opportunistic human pathogen *Pseudomonas aeruginosa* PAO1 and the plant pathogen *Pseudomonas syringae* DC3000 have been performed [27], [28], [29]. In both organisms, the transcription of more than 300 genes was significantly influenced by iron [27], [28], [29]. Despite the importance of rhizospheric bacterial biocontrol agents, no studies have investigated the global effects of iron limitation in these organisms. Therefore, this study was undertaken to observe the global transcriptomic and proteomic changes in Pf-5 when faced with iron limitation. Here, we observed a wide ranging physiological effect on *P. fluorescens* Pf-5 by iron limitation, including changes to the expression of important biocontrol factors.

## Results and Discussion

### Growth of Pf-5 in Iron-limited and Iron-amended Media

Iron commonly occurs in the forms of two oxidation states, i.e., the +2 and +3 oxidative states. In the presence of oxygen and when pH is above 5, iron(II) will oxidize rapidly to iron(III) in a solution [30]. Nevertheless, different physiological consequences of FeCl_2_ and FeCl_3_ supplementation on cell cultures grown under oxygenic conditions have been observed, such as in a study on eukaryotic Vero monkey kidney cells [31]. Additionally, a study with *Pseudomonas putida* conducted by Molina et al. [32], showed that overnight cultures supplemented with either FeCl_2_ or FeCl_3_ had different growth biomass, which might suggest different levels of iron bioavailability or chemistry of the two iron sources used. Therefore, we employed both FeCl_2_ and FeCl_3_ in our study to observe whether usage of these two iron sources has any differential effects on Pf-5 at the molecular level, besides looking at the effect of iron limitation of *P. fluorescens* Pf-5.

The growth rate of *P. fluorescens* Pf-5 was higher in M9 minimal medium supplemented with either 100 µM FeCl_2_ or 100 µM FeCl_3_ than in non-amended M9 medium. Additionally, the total cell biomass was higher during stationary phase in both FeCl_2_- and FeCl_3_-amended cultures than in non-amended medium (Figure S1). Unlike observations reported for *P. putida* [32], we observed no notable difference between the growth rates and final cell densities of Pf-5 cultures supplemented with equal amounts of FeCl_2_ versus FeCl_3_ under our conditions (Figure S1). In the non-amended medium, Pf-5 produced a yellow-green fluorescent pigment characteristic of pyoverdine production.

### General Assessment of Iron Limitation Microarray Datasets

**Figure 1:**
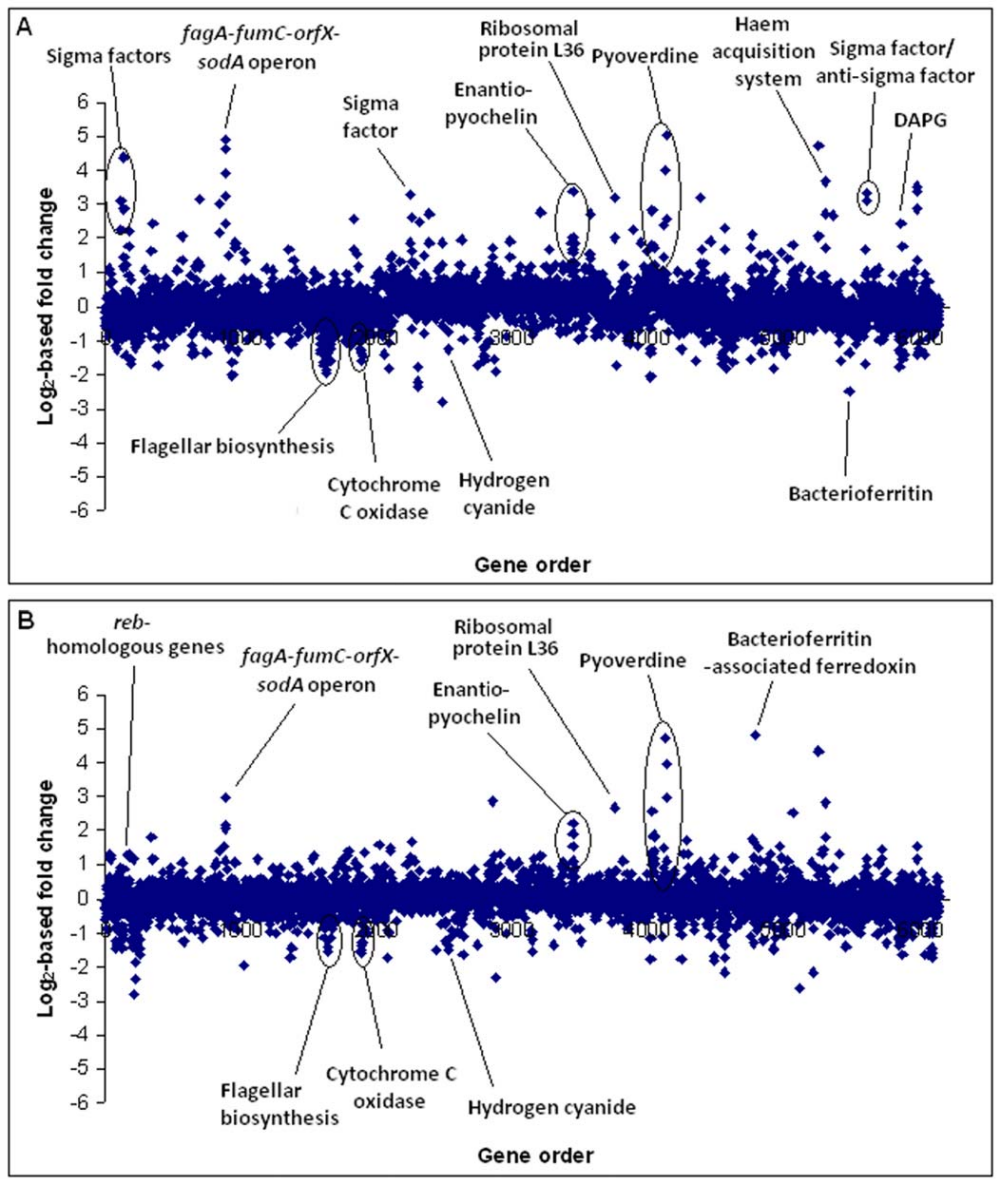
Differential gene transcription in cells grown in an iron-limited medium compared to iron-supplemented media . The charts show log_2_-based fold changes of transcript of cells grown in an iron-limited medium compared to a medium supplemented with either (A) FeCl_2_ or (B) FeCl_3_. Each dot in the chart represents one of 6147 annotated genes in the Pf-5 genome with the *x*-axis showing gene order (the origin of replication at the far left and far right), and the *y*-axis showing the log_2_ of relative transcript abundance for each gene in cells of Pf-5 grown in an iron-limited medium relative to the iron-amended medium.

The transcription of 180 and 121 genes increased or decreased by at least 2-fold, respectively, in iron-starved cells as compared to cells receiving FeCl_2_ supplementation (Figure 1A; Table S1). Transcription of only 38 and 86 genes increased or decreased by at least 2-fold, respectively, in iron-starved cells versus cells grown in medium supplemented with FeCl_3_ (Figure 1B; Table S1). Many of the genes undergoing transcriptional changes were known or predicted to be involved in iron homeostasis, allowing the delineation of the genes encoding iron uptake machinery in Pf-5. There was significant overlap between genes transcriptionally regulated in the two experiments although a number of genes involved in iron homeostasis (i.e., PFL_0909, PFL_3496, PFL_4080 and PFL_5555) had significantly higher transcriptional response upon FeCl_2_ supplementation versus FeCl_3_ supplementation, as discussed below (Table S1). The disparity in transcriptional levels observed between the experiments might be due to differences in iron bioavailability or chemical properties of the iron sources used.

The genes found in the Pf-5 genome have been divided into 18 functional categories (http://cmr.jcvi.org/cgi-bin/CMR/shared/RoleList.cgi). Under iron-limited conditions, genes in the role categories of “transport and binding proteins” and “regulatory function” were notably up-regulated (Figure S2). In the category for “regulatory function”, many of the genes consisted of extra-cytoplasmic function (ECF) sigma factors that are adjacent to TonB-dependent receptor genes that may function in iron uptake (see below) [17].

qRT-PCR validation was performed on the microarray data using a subset of 22 differentially regulated genes (Table S2). In experiments utilizing FeCl_2_ as the iron source, the microarray data generally corresponded well with qRT-PCR data with a Pearson correlation coefficient of 0.86 (Figure S3). Minor discrepancies were observed between the qRT-PCR and microarray results for three genes (PFL_1649, PFL_4410 and PFL_5965). Such occurrences are not unique and several other studies observed similar discrepancies [33], [34]. In the case of FeCl_3_, a good correlation was observed, with a Pearson correlation coefficient of 0.93 (Figure S3).

### Iron Limitation Increased Transcription of Iron Acquisition Systems

Genes for the biosynthesis and uptake of the siderophores pyoverdine and enantio-pyochelin were more highly expressed in iron-limited cultures of Pf-5 than in iron-amended cultures (Figure 1). Within the up-regulated pyoverdine biosynthesis gene clusters, the most highly expressed gene was *pvdS* (PFL_4190), which encodes the extra-cytoplasmic function (ECF) sigma factor PvdS, a transcriptional regulator of pyoverdine biosynthesis genes (Table 1). Recently, Hassan et al. [35] identified putative PvdS-controlled promoter regions upstream of 16 genes in the pyoverdine biosynthesis gene clusters of Pf-5 based on their similarity to the PvdS-controlled promoters of *P. syringae* [25] and *P. aeruginosa* [27]. In this study, all genes directly downstream of a putative PvdS-controlled promoter region were transcriptionally up-regulated, although not always by more than 2-fold (data not shown). Within the enantio-pyochelin gene cluster (PFL_3488-3497), the gene most highly regulated by iron was *pchR* (PFL_3497), an AraC family regulator that controls transcription of genes for the biosynthesis and transport of enantio-pyochelin [36] (Table 1). Genes (PFL_3498 to PFL_3503) adjacent to the currently defined enantio-pyochelin cluster were also up-regulated in iron-limited medium (Table 1). These genes encode proteins with predicted functions in membrane transport and may have roles in the uptake of iron-bound enantio-pyochelin [16], [36].

**Table 1:** Transcriptional regulation by iron of genes for siderophore biosynthesis and transport.

		Fold change (log_2_)	
Genes	Annotated functions	Iron-deprived (-FeCl_2_)	Iron-deprived (-FeCl_3_)
	**Pyoverdine biosynthesis gene cluster**		
PFL_2901	hypothetical protein	**1.71**	NS
PFL_4079	L-ornithine 5-monooxygenase PvdA	**1.81**	NS
∧FL_4080	RNA polymerase sigma-70 factor, ECF subfamily, FpvI	**2.84** *	**1.02** *
PFL_4082	efflux ABC transporter, ATP-binding/permease protein	**1.09**	NS
PFL_4083	efflux transporter, outer membrane factor lipoprotein, NodT family	**1.09**	NS
PFL_4086	chromophore maturation protein PvdP	**1.15**	NS
PFL_4093	non-ribosomal peptide synthetase PvdD	**1.74**	NS
PFL_4095	non-ribosomal peptide synthetase PvdI	**1.19**	NS
PFL_4096	siderophore-interacting protein	**1.17**	**1.17**
PFL_4169	PepSY-associated TM helix domain protein	**2.39**	**1.48**
PFL_4171	conserved hypothetical protein	**1.44**	NS
PFL_4178	MbtH-like protein	**3.99**	**4.73**
∧FL_4189	non-ribosomal peptide synthetase PvdL	**2.59**	NS
∧FL_4190	polymerase sigma-70 factor, ECF subfamily, PvdS	**5.07**	**3.94**
	**Enantio-pyochelin biosynthesis gene cluster**		
∧FL_3483	RNA polymerase sigma-70 factor, ECF subfamily	**1.87**	NS
PFL_3484	sigma factor regulatory protein, putative	**1.14**	NS
PFL_3490	enantio-pyochelin biosynthetic protein PchC	**1.21**	NS
PFL_3491	saccharopine dehydrogenase PchK	**1.30**	NS
PFL_3492	enantio-pyochelin synthetase PchF	**1.11**	NS
PFL_3495	ABC transporter, permease/ATP-binding protein, putative, PchH	**1.60**	NS
PFL_3496	salicyl-AMP ligase PchD	**1.11** *	0.45*
PFL_3497	regulatory protein PchR	**3.41**	**1.53**
PFL_3498	TonB-dependent outermembrane enantio-pyochelin receptor FetA	**2.01**	NS
PFL_3499	PepSY-associated membrane protein	**1.70**	NS
PFL_3500	iron-chelate uptake ABC transporter, FeCT family, periplasmic iron-chelate-binding protein, putative	**1.87**	NS
PFL_3501	iron-chelate uptake ABC transporter, FeCT family, permease protein	**1.23**	NS
PFL_3502	iron-chelate uptake ABC transporter, FeCT family, ATP-binding protein	**1.23**	NS
PFL_3503	transporter, major facilitator family	**1.06**	NS

∧Pf-5 genes that contain Fur binding motifs upstream [35].

* Values are statistically different as determined using MeV software with P-value <0.01. NS means not significant when analyzed with SAM at FDR <1% in this study. Numbers in bold denote fold changes that surpass the significant differential expression threshold defined in this study, i.e. equal to or exceeding 2-fold change for transcript levels.

In addition to siderophores, Pf-5 has haem acquisition systems encoded by three distinct clusters within the genome (PFL_4625-29, PFL_5263-66 and PFL_5374-80) [35], [37]. The *hasA* gene (PFL_5377), which encodes a protein homologous to a haemophore produced by *P. aeruginosa* [37], and *hasI* (PFL_5380), which encodes an ECF sigma factor, were highly up-regulated in iron-limited medium (Table 2). Pf-5 also has genes for an alternative haem uptake system that is homologous to the *phu* operon of *P. aeruginosa* [38]. In the *phu* operon of Pf-5, the genes encoding a sigma factor (PFL_4625) and haem oxygenase (PFL_4628, *hemO*) as well as a putative haem ABC transporter (PFL_5266, *phuT*) were up-regulated by more than 2-fold under iron-limited conditions (Table 2). The haem oxygenase is involved in haem degradation to release the bound iron [39]. Its orthologues in *P. aeruginosa* and *P. syringae* were also highly up-regulated when iron was limited [27], [29] (Table S1).

**Table 2:** Transcriptional regulation by iron of genes for iron acquisition, oxidative stress response and iron storage.

		Fold change (log_2_)	
Genes	Annotated functions	Iron-deprived (-FeCl_2_)	Iron-deprived (-FeCl_3_)
	**Haem acquisition systems**		
PFL_4625∧	RNA polymerase sigma-70 factor, ECF subfamily	**1.70**	0.85
PFL_4628	heme oxygenase HemO	**2.28**	NS
PFL_5266∧	hemin ABC transporter, periplasmic hemin-binding protein PhuT	**1.01**	0.37
PFL_5377	heme acquisition protein HasAp	**3.70**	NS
PFL_5379	sigma factor regulatory protein HasS	**2.74**	NS
PFL_5380∧	RNA polymerase sigma-70 factor, ECF subfamily, HasI	**1.72**	NS
	**Extra-cytoplasmic sigma factors and TonB-dependent receptors**		
PFL_1373	RNA polymerase sigma-70 factor, ECF subfamily	**1.68**	0.70
PFL_3156	RNA polymerase sigma-70 factor, ECF subfamily	**1.05**	NS
	**TonB-dependent receptors**		
PFL_0932	TonB-dependent receptor	**1.49**	NS
PFL_3177	TonB-dependent outer membrane receptor	**1.14**	NS
PFL_4912	TonB-dependent outer membrane receptor	NS	**−1.21**
	**ExbB, ExbD and TonB**		
PFL_0225	TonB2 protein	NS	**−2.83**
PFL_0226	TonB system transport protein ExbB2	−0.99	**−2.35**
PFL_6067	TonB system transport protein ExbB1	**3.40**	NS
PFL_6068	TonB system transport protein ExbD1	**2.85**	NS
PFL_6069	periplasmic energy transduction protein TonB1	**3.55**	**1.52**
	**Iron transport across inner membrane**		
PFL_0573	iron ABC transporter permease FbpB	**1.35**	NS
PFL_0574	iron ABC transporter substrate-binding protein FbpA	**2.08**	**1.06**
PFL_0910	zinc(II)-iron(II) family metal cation transporter permease	**2.44**	NS
PFL_3255	ferrous iron permease EfeU	**2.76**	**1.25**
PFL_5964∧	ferric iron ABC transporter, FeT family, periplasmic ferric iron-binding protein, putative	**1.78**	NS
	**Oxidative stress response and iron storage**		
PFL_0909	superoxide dismutase, Mn, SodA1	**3.27** *	**1.13** *
PFL_4826	superoxide dismutase (Fe) SodB	**−1.53**	**−1.13**
PFL_4858∧	bacterioferritin-associated ferredoxin, putative	NS	**4.83**
PFL_5555	bacterioferritin A	**−2.48** *	−0.72*
PFL_5556	catalase KatA	**−1.37**	−0.70

∧Pf-5 genes that contain Fur binding motifs upstream [35].

* Values are statistically different as determined using MeV software with P-value <0.01. NS means not significant when analyzed with SAM at FDR <1% in this study. Numbers in bold denote fold changes that surpass the significant differential expression threshold defined in this study, i.e. equal to or exceeding 2-fold change for transcript levels.

Motifs characteristic of Fur binding sites, which strongly resemble the Fur binding site of *P. syringae* [20], were identified between 14 and 209 nt 5′ of predicted translational start sites for 38 genes in the Pf-5 genome [35], including three genes in the pyoverdine biosynthesis region (PFL_4080, *fpvI*; PFL_4189, *pvdL*; and PFL_4190, *pvdS)* and two genes involved in haem uptake (PFL_5378, *hasR*; and PFL_5380, *hasI*). Of these 38 genes, transcripts of 27 were significantly up-regulated in iron-limited culture medium (Table S3). Similar to iron limitation studies in *P. aeruginosa* [27], [28] and *P. syringae* [20], [29], we observed that some genes encoded downstream of putative Fur binding motifs (e.g. PFL_2491 encoding a TetR regulator) were not iron-regulated, possibly because other factors controlling their transcription may counteract and obscure the effect of iron under the conditions of this study [40].

### Transcriptional Effects on Extra-cytoplasmic Sigma Factors and TonB-dependent Receptors

The genome of Pf-5 includes 28 genes predicted to encode ECF sigma factors, many of which are likely to control iron homeostasis [6]. 18 of the genes encoding these ECF sigma factors are situated adjacent to or near genes encoding anti-sigma factors and TonB-dependent receptors that contain N-terminal signaling domains. Upon receiving an external signal, the N-terminal signaling domains of these receptors will interact with anti-sigma factors, which then release the bound sigma factors thereby allowing the sigma factors to participate in the transcription of target genes [41]. These regulatory systems are involved in cell-surface signaling, which bacteria use to detect signals from the environment and transmit them into the cytoplasm [41]. Of the 18 sigma factor genes described above, 13 have consensus Fur binding sites upstream from their translational start sites that are likely to control their transcription [35] (Table S4). A Fur binding site is also upstream of the gene encoding the FpvI sigma factor (PFL_4080), which is unusual as its cognate anti-sigma factor (PFL_2903, *fpvR*) and TonB-dependent receptor for pyoverdine (PFL_4092) are located distally within the genome [17]. All 14 sigma factor genes having upstream Fur binding sites were up-regulated under the iron-limited conditions (Table S4). While no Fur binding site was detected upstream of five other genes encoding ECF sigma factors that are clustered with anti-sigma factor and TonB-dependent receptor genes, two of the sigma factor genes (PFL_1373 and PFL_3156) were up-regulated in iron-limited media (Table 2).

There are 45 predicted TonB-dependent receptors in Pf-5, 27 of which lack an N-terminal signaling domain [17]. Some of these TonB-dependent receptors are predicted transporters for heterologous ferric-siderophore complexes [17]. In this study, some TonB-dependent receptors that lack an N-terminal signaling domain were up-regulated (PFL_0932, PFL_3177, PFL_3498) or down-regulated (PFL_4912) in the iron-limited media (Tables 1 and 2). No Fur boxes were found upstream of these genes [35]. PFL_3498 (*fetA*) encodes the enantio-pyochelin receptor [42], but substrates for the other receptors are not known.

TonB-dependent receptors rely on the accessory proteins ExbB, ExbD and TonB for energy transduction [43]. Two of the five annotated *tonB-exbB-exbD* clusters in the Pf-5 genome were regulated by iron; PFL_0225-27 was significantly down-regulated in iron-limited cultures (when compared to cells grown in culture supplemented with FeCl_3_) and PFL_6067-69 was significantly up-regulated. *P. aeruginosa* PAO1 encodes a *tonB2-exbB-exbD* locus PA0197-99, which is orthologous to PFL_0225-27 of Pf-5 (Table 2). This cluster has not been reported to be iron-regulated but is strongly up-regulated under sulfate starvation conditions [33]. Cornelis et al. [19] suggest that there is an overlap between the iron and sulfur regulons in *P. aeruginosa*. Because PFL_6067-69, which has no orthologous cluster in PA01, was the only e*xbB-exbD-tonB* cluster significantly up-regulated in cells of Pf-5 grown in iron-limited culture medium, it is likely to function in iron acquisition. The orthologous cluster in *P. syringae* was also transcriptionally up-regulated under iron-limited conditions [29] (Table S1).

### Transcription of Genes Involved in Iron Transport Across Inner Membrane

In addition to ferric-siderophore uptake systems, other mechanisms are employed by bacteria to take up iron(II) and iron(III) in a free form. In anaerobic or reducing conditions, iron(II) is stable and soluble and can freely enter bacterial cells through the outer membrane porins of gram-negative bacteria [37]. Once in the periplasm, iron(II) is taken across the inner membrane by divalent metal uptake transporters [44]. Haritha et al. [45] performed a bioinformatic analysis, utilizing the TransportDB database [46], to identify the metal transportome of Pf-5. One of the predicted metal transporters is a gene encoding a zinc(II)-iron(II) permease family metal cation transporter (PFL_0910), which was significantly over-transcribed under iron limitation condition in our study, suggesting a role in the uptake of free iron (Table 2).

Many bacteria have the capacity to transport ferrous iron into the cytoplasm, and homologues of EfeUOB of *Escherichia coli,* which facilitates uptake of iron(II) in acidic conditions [47], are present in many bacterial genera. The PFL_3255 membrane transporter, which was described by Haritha et al. [45] as an iron transporter, has 58% identity to the EfeU protein of *E. coli* K12. In our study, this gene was up-regulated strongly under iron limiting conditions (Table 2). A similar effect was observed for its orthologue in *P. syringae* [29] (Table S1). In *E. coli*, this operon is regulated by Fur and induced by acidic conditions [47]. A previous analysis of the Pf-5 genome did not detect a Fur box upstream of the gene [35] but this binding motif was found when a HMM search was performed with HMMER2 [48] at a lower E-value parameter of 1e-1 (data not shown).

Several genes encoding components of ABC transport systems predicted to be involved in iron(III) uptake across the cytoplasmic membrane were highly transcribed under the iron limiting condition used in our study. These include the genes located in PFL_5963-64 and PFL_0573-74 operons, which are homologous to the well-characterized iron(III) transporters HitABC of *Haemophilus influenzae* [49] and SfuABC of *Serratia marcescens* [50], respectively (Table 2). In concordance, transcription of the orthologous genes in *P. aeruginosa* is enhanced under iron-limited conditions [27], [28] (Table S1).

### Oxidative Stress Response and Iron Storage

Bacterial oxidative stress responses are associated with iron homeostasis due to the role of iron in production of reactive oxygen species such as superoxide (O_2_-) and hydroxyl radicals (HO.) [51]. The presence of iron(II) inside the cell can result in the production of HO. through the Fenton reaction (iron(II) + H_2_O_2_ → iron(III) + HO.). To counter this, excess cellular iron(II) is converted to iron(III) through ferroxidase activity imparted by bacterioferritins and stored as ferric-oxy-hydroxide-phosphate complexes within bacterioferritins [52]. In the iron-limited medium, PFL_5555, which is orthologous to a *P. aeruginosa* gene encoding a non-haem binding bacterial ferritin (PA4235) [53], was down-regulated in Pf-5 (Table 2). Similar results were also observed in *P. syringae*, possibly because the need for iron storage was reduced under low-iron conditions [29] (Table S1). In addition, a bacterioferritin-associated ferredoxin gene (PFL_4858) was up-regulated by Pf-5 in iron-limited medium, as observed previously in *P. aeruginosa* [27], [28] and *P. syringae* [29] (Table 2; Table S1). The bacterioferritin-associated ferredoxin mobilizes iron stored in bacterioferritin B, and PFL_4858 gene is adjacent to a bacterioferritin B-encoding gene (PFL_4859), implying their cooperative nature [54].

Transcriptional changes were also observed for genes encoding oxidative stress response proteins such as superoxide dismutases, which counter reactive oxygen species by converting O_2_^−^ to H_2_O_2_. In Pf-5, a gene (PFL_4826) coding for a superoxide dismutase that utilizes iron as cofactor (SodB) was down-regulated in iron-limited conditions, presumably due to the reduced availability of iron in the cells (Table 2). Conversely, a manganese-based superoxide dismutase (PFL_0909, *sodA*) was up-regulated. This gene is part of the *fagA-fumC-orfX-sodA* operon (PFL_0906-09) that is involved in oxidative stress responses in *P. aeruginosa* [55], [56], [57]. This operon may be regulated by Fur, since it is encoded downstream of a putative Fur binding motif [35]. PFL_5556, a homologue of PA4236, which encodes the KatA catalase of *P. aeruginosa*, was down-regulated by iron limitation (Table 2). This gene is adjacent to the bacterioferritin A gene (PFL_5555) in the Pf-5 genome, which was also down-regulated under iron-limited conditions, as described above. The genetic organization of the two genes is similar in Pf-5 and *P. aeruginosa* PAO1, where the genes are known to be expressed from different transcripts [52]. In *P. aeruginosa*, a functional bacterioferritin A is critical to the catalase activity of KatA [52]. Similarly, the regulation of *katA* and bacterioferritin A could be linked in Pf-5.

### Transcription of Respiratory Chain Genes

*Pseudomonas* spp. have extensively branched respiratory chain systems with multiple terminal cytochrome oxidases, thereby possessing the flexibility to use the electron transfer chain that is most suitable for the specific environmental conditions encountered [58], [59]. In aerobic metabolism, the respiratory chain typically uses proteins that require iron cofactors [27]. When Pf-5 was grown in the iron-limited medium, genes encoding cytochrome c-type biogenesis proteins (PFL_1684-88) and subunits of *cbb3*-type cytochrome c oxidases (PFL_1922-25, PFL_2834) were down regulated (Table 3; Table S1), whereas genes encoding subunits of a second *cbb3*-type cytochrome c oxidase (PFL_1918-1921) were not regulated significantly by iron. Similarly, in *P. aeruginosa*, transcription of the *cbb3*-type cytochrome c oxidase subunit II gene (PA1556), but not its paralogous gene (PA1553), is lower in an iron-limited versus an iron-replete medium [28]. In contrast, transcription of genes encoding subunits of a cytochrome *o* ubiquinol oxidase (PA1317-21, *cyoABCDE*) increased under iron limitation in *P. aeruginosa* [27], [59]. In Pf-5, only one gene (PFL_5037) in the *cyoABCDE* cluster (PFL_5033-37) was up-regulated in the iron-limited medium, and that effect was observed only in the experiment evaluating the influence of FeCl_3_ (Table 3). Transcription of a second *cyoABCD* cluster (PFL_4732-35) was not influenced by iron. Several other genes that encode participants in the electron transport chain, such as those coding for ubiquinol-cytochrome *c* reductase (PFL_5078-80), cytochrome *c*4 (PFL_0084) and a cytochrome *c* family protein (PFL_5004), were down-regulated in cells grown under iron limitation (Table 3). As described for other bacteria [60], [61], iron availability appears to alter the preferred branch of the electron chain utilized by Pf-5 growing in an aerobic environment, providing a mechanism for the bacterium to conserve iron demands on the cell.

**Table 3:** Transcriptional regulation by iron of genes involved in respiratory chain components and flagellar biosynthesis.

		Fold change (log_2_)	
Genes	Annotated functions	Iron-deprived (-FeCl_2_)	Iron-deprived (-FeCl_3_)
	**Respiratory chain gene clusters**		
PFL_0084	cytochrome c4	**−1.18** *	−0.46*
PFL_1684	cytochrome c-type biogenesis protein CcmE	**−1.24**	NS
PFL_1685	cytochrome c-type biogenesis protein CcmF	**−1.04**	NS
PFL_1687	cytochrome c-type biogenesis protein CycL	**−1.10**	NS
PFL_1917	cytochrome c oxidase accessory protein CcoG	**−1.58**	−0.72
PFL_1922	cytochrome c oxidase, cbb3-type, subunit III, CcoP_2	NS	**−1.29**
PFL_1923	cytochrome c oxidase, cbb3-type, CcoQ subunit	**−1.00**	**−1.36**
PFL_1924	cytochrome c oxidase, cbb3-type, subunit II, CcoO_2	**−1.57**	**−1.47**
PFL_1925	cytochrome c oxidase, cbb3-type, subunit I, CcoN_2	NS	**−1.57**
PFL_2834	cytochrome c oxidase, cbb3-type, subunit I, CcoN_3	**−1.57**	−0.74
PFL_5004	cytochrome c family protein	**−1.18**	**−1.17**
PFL_5037	cytochrome o ubiquinol oxidase, subunit II, CyoA_2	NS	**1.55**
PFL_5078	ubiquinol-cytochrome c reductase, cytochrome c1, putative	**−1.20**	NS
PFL_5080	ubiquinol-cytochrome c reductase, iron-sulfur subunit PetA	**−1.41**	NS
	**Flagellar biosynthesis gene clusters**		
PFL_1636	sigma-54 dependent DNA-binding response regulator FleR	**−1.41**	**−1.16**
PFL_1637	flagellar hook-basal body complex protein FliE	**−1.66**	NS
PFL_1638	flagellar M-ring protein FliF	**−1.74** *	−0.87*
PFL_1639	flagellar motor switch protein FliG	**−1.75** *	−0.70*
PFL_1640	Flagellar assembly protein FliH	**−1.18**	NS
PFL_1641	flagellum-specific ATP synthase FliI	**−1.12**	NS
PFL_1646	flagellar hook-length control protein FliK	**−1.23**	NS
PFL_1647	flagellar protein FliL	**−1.38**	NS
PFL_1648	flagellar motor switch protein FliM	**−1.47**	NS
PFL_1649	flagellar motor switch protein FliN	**−1.96**	NS
PFL_1652	flagellar biosynthetic protein FliQ	**−1.04**	NS
PFL_1656	conserved hypothetical protein	**−1.87**	NS
PFL_1657	3-oxoacyl-(acyl-carrier-protein) synthase III, putative	**−1.32**	−0.68
PFL_1664	flagellar biosynthesis protein FlhA	**−1.48**	NS
PFL_1666	flagellar synthesis regulator FleN	NS	**−1.55**
PFL_1667	motility sigma factor FliA	NS	**−1.39**
PFL_4480	flagellar basal-body rod protein FlgB	**−1.08**	NS

* Values are statistically different as determined using MeV software with P-value <0.01. NS means not significant when analyzed with SAM at FDR <1% in this study. Numbers in bold denote fold changes that surpass the significant differential expression threshold defined in this study, i.e. equal to or exceeding 2-fold change for transcript levels.

### The Effect of Iron Limitation on Swarming Motility

**Figure 2:**
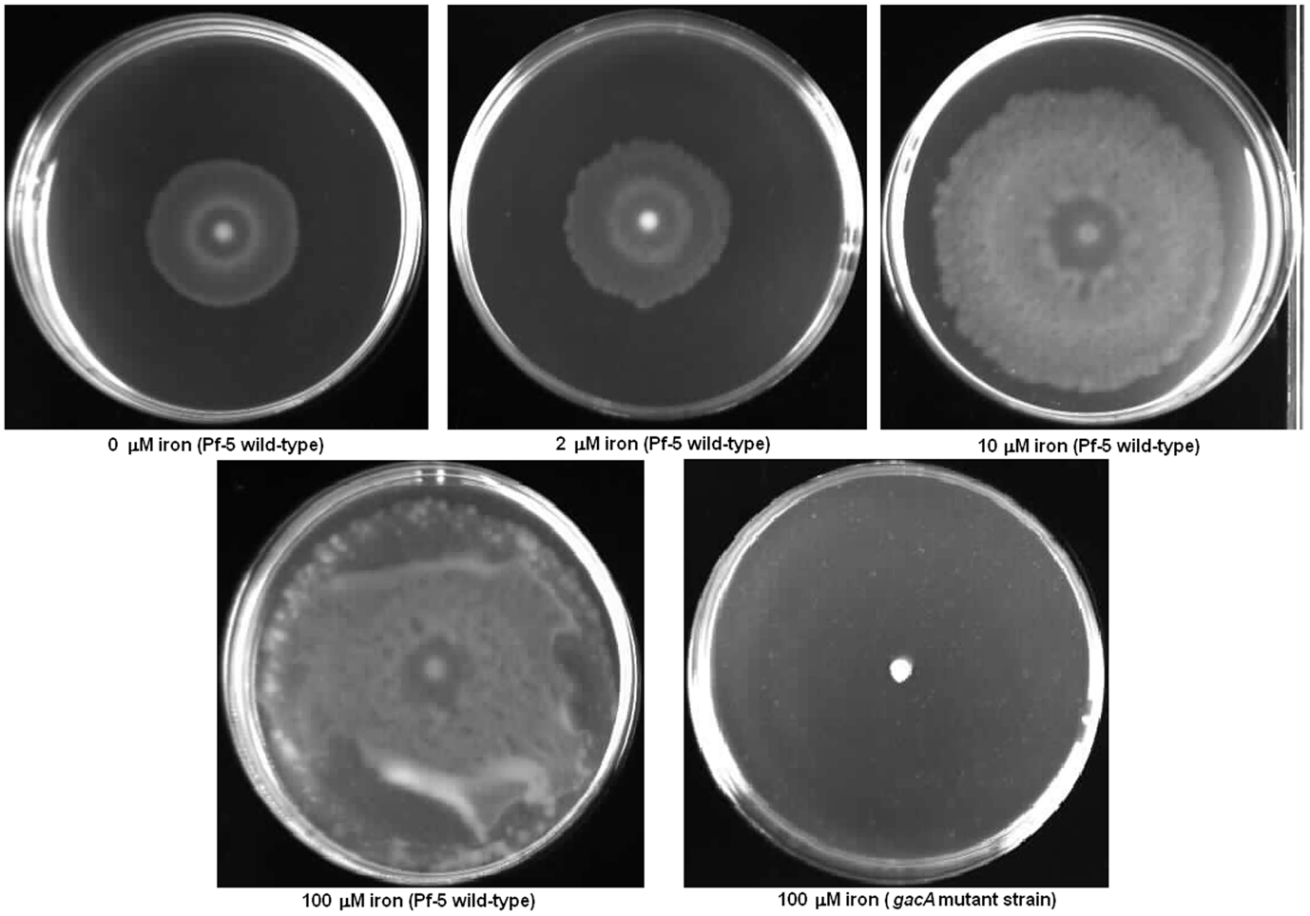
Iron concentration-dependent swarming motility Swarming motility of strain Pf-5 and a *gacA* mutant, which served as swarming-deficient control, was observed on M9 minimal medium containing 0.6% agar supplemented with varied concentrations of FeCl_2_ as indicated below each panel.

Iron limitation generally resulted in reduced transcription of some genes in the flagellar biosynthesis clusters (PFL_1629-73 and PFL_4477-85) (Table 3). For example, transcription of *fliA* (PFL_1667) was reduced in the iron-limited culture compared to the FeCl_3_-supplemented culture. The *fliA* gene encodes a sigma factor controlling flagellar biosynthesis and motility in *P. aeruginosa* [62] and its reduced expression in Pf-5 may account for the decreased expression of flagellar biosynthesis genes. We evaluated swarming motility of Pf-5 on 0.6% agar plates containing M9 minimal media supplemented with varied concentrations of iron, and observed that the diameters of the swarming colonies were proportional to iron concentration of the medium (Figure 2). Our observation is in contrast to previous reports that motility of *P. aeruginosa*, *P. syringae* and *P. putida* is promoted by iron limitation [63], [64], [65], [66]. It was suggested that this phenomenon is a chemotactic response of *P. aeruginosa*, allowing it to migrate to another location in search of nutrients [64], [67]. *P. aeruginosa* did not exhibit transcriptional changes in genes involved in flagellar biosynthesis under conditions of iron limitation [27], [28], which suggests that transcriptional regulation of the genes differs between the species.

### Iron Limitation Affects Transcription of Ribosomal Protein L36 Gene

A ribosomal protein L36 gene (PFL_3806) was highly up-regulated under iron-limited conditions in Pf-5 (Table 4). Interestingly, a putative Fur binding site was observed upstream of the gene [35]. A second non-annotated ribosomal protein L36 gene was identified at coordinates 6351985-6351869 in Pf-5 (RefSeq number: NC_004129.6). In *P. aeruginosa*, two paralogues of ribosomal protein L36 (C+ and C- forms) have been found [68]. The L36 C+ form has a ‘CXXC.CXXXH’ motif that forms a metal-binding zinc-ribbon motif [69], which is absent in the C- form. PFL_3806 corresponds to the C- protein. The transcription of ribosomal protein L36-encoding genes was not significantly affected by iron limitation in *P. aeruginosa* or *P. syringae* [27], [28], [29]. The reason for the high-level transcription of this gene by Pf-5 under iron-limited conditions remains to be answered. The gene product could be involved in iron homeostasis in Pf-5, reminiscent of the functions of some ribosomal proteins in zinc homeostasis in other bacterial species [28].

**Table 4:** Transcriptional regulation by iron of selected genes.

		Fold change (log_2_)	
Genes	Annotated functions	Iron-deprived (-FeCl_2_)	Iron-deprived (-FeCl_3_)
	**Ribosomal protein L36 gene**		
PFL_3806∧	ribosomal protein L36 RpmJ	**3.20**	**2.67**
	***reb*** **-homologous genes**		
PFL_0183	RebB protein	0.91	**1.25**
PFL_0184	RebB protein	NS	**1.31**
	**Secondary metabolite biosynthesis**		
PFL_2143	transcriptional regulator, LuxR family	NS	**1.48**
PFL_2147	Nonribosomal peptide synthetase OfaC	**1.41**	NS
PFL_2577	hydrogen cyanide synthase HcnA	NS	**−1.49**
PFL_2578	hydrogen cyanide synthase HcnB	**−1.26**	**−1.49**
PFL_2579	hydrogen cyanide synthase HcnC	NS	**−1.36**
PFL_5953	transcriptional repressor PhlF	**2.42**	NS
PFL_5954	2,4-diacetylphloroglucinol biosynthesis protein PhlA	**2.44**	NS

∧Pf-5 genes that contain Fur binding motifs upstream [35]. NS means not significant when analyzed with SAM at FDR <1% in this study. Numbers in bold denote fold changes that surpass the significant differential expression threshold defined in this study, i.e. equal to or exceeding 2-fold change for transcript levels.

### *reb*-homologous Genes were Highly Transcribed under Iron Limitation

Two genes (PFL_0183 and PFL_0184) that were highly transcribed by Pf-5 grown in the iron-limited medium (Table 4) are similar to *reb* genes of *Caedibacter taeniospiralis* [70], which putatively encode refractile inclusion bodies (R-bodies). R-bodies are cylindrical structures in the bacterial cell, which are composed of insoluble proteinaceous ribbons and are associated with toxicity [71], [72]. For example, the R-bodies produced by *C. taeniospiralis* are thought to kill a sensitive paramecium [73]. R-bodies have been observed in *P. aeruginosa* [74] and other pseudomonads, such as *Pseudomonas taeniospiralis* [75] and *Pseudomonas avenae* [76], but have not been observed in *P. fluorescens*. Of the four published *P. fluorescens* genomes (Pf-5, SBW25, Pf0-1 and WH6), only Pf-5 has genes predicted to encode R-bodies (PFL_0180, PFL_0183 and PFL_0184) [6], [77], [78]. The synthesis of R-bodies has been associated with mobile elements such as plasmids and prophages [71], [72], but there is no evidence of recent horizontal acquisition of the *reb* genes by Pf-5.

### Effect of Iron Limitation on Transcription of Genes Involved in Secondary Metabolite Biosynthesis

Iron is known to influence the production of secondary metabolites by *Pseudomonas* spp., such as the phytotoxins syringomycin and syringotoxin produced by *P. syringae* [79], [80]. Accordingly, transcription of many secondary metabolite biosynthesis genes by Pf-5 was influenced by iron amendment of the medium. As shown in a previous study by Blumer and Haas [81], hydrogen cyanide biosynthesis genes (PFL_2577-79) were down-regulated in iron-limited medium (Figure 1; Table 4; Table S1). Blumer and Haas [81] suggested that the ANR regulator, which controls hydrogen cyanide production by binding to the promoter of the *hcnABC* cluster, is sensitive to iron concentration as it contains Fe-S clusters. A lack of iron restricts the assembly of the Fe-S clusters on the regulator, potentially disabling its function. Within the DAPG biosynthesis cluster, genes encoding the transcriptional repressor PhlF (PFL_5953) and the biosynthetic enzyme PhlA (PFL_5954) were up-regulated in Pf-5 grown in the iron-limited medium (Table 4). Within the orfamide A biosynthesis cluster, genes encoding a transcriptional regulator of the LysR family (PFL_2143) and the non-ribosomal peptide synthetase OfaC (PFL_2147) were up-regulated in the iron-limited medium (Table 4). We did not observe significant, 2-fold or greater, transcriptional effects of iron on other biosynthetic genes within the other known secondary metabolic gene clusters in Pf-5 (i.e., pyoluteorin, pyrrolnitrin, or rhizoxin analogs).

### Comparison with Pf-5 *gacA* Mutant Strain Transcriptional Profile

**Figure 3:**
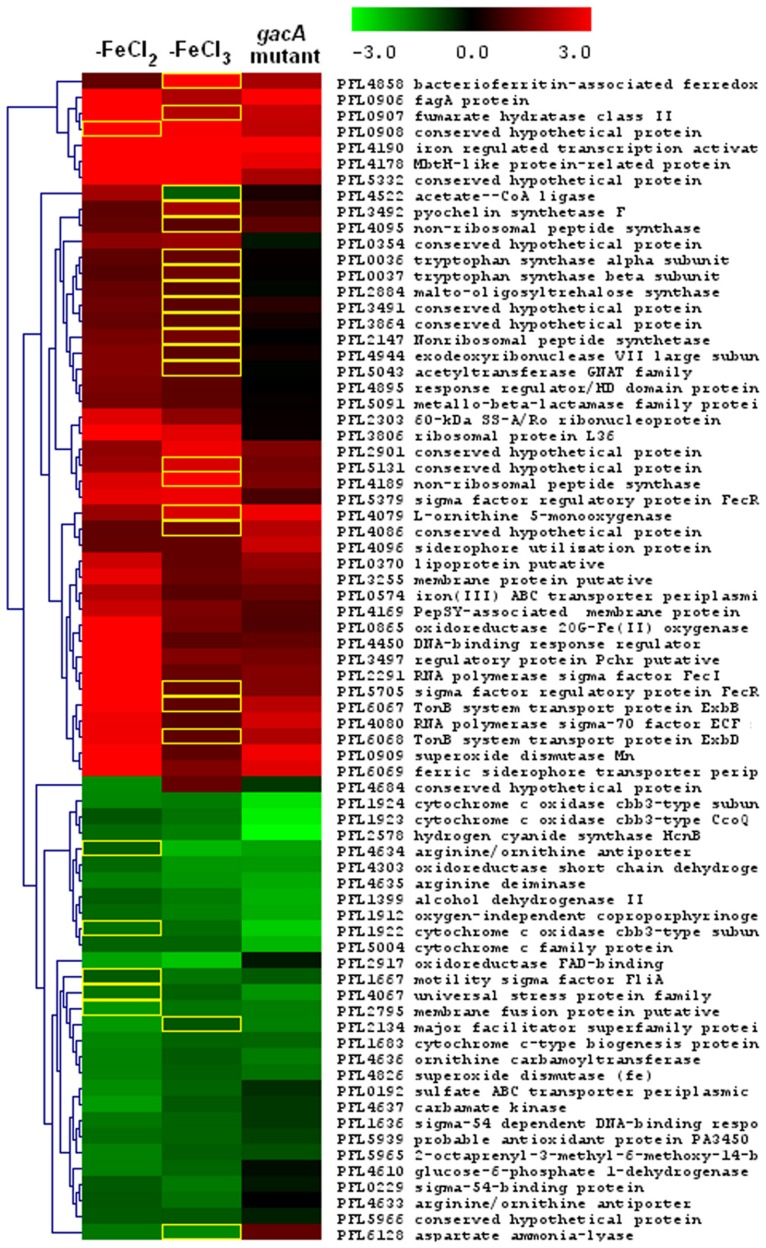
Heat map of hierarchical analysis of genes differentially regulated by iron . In addition to data derived from this iron limitation study, microarray data on transcriptional fold changes between a *gacA* mutant and the parental strain Pf-5, from study by Hassan et al. [35], was included for comparison. This map was constructed by average linkage clustering analysis with Euclidean distance matrix in MeV software [110]. We included only those genes that were regulated by more than 2-fold by iron in this study. Genes that were regulated significantly by iron (with an FDR of less than 5%) are indicated as yellow boxes. -FeCl_2_ and -FeCl_3_ respectively represent results obtained when iron-limited cultures were compared to FeCl_2_ and FeCl_3_ supplemented cultures. Red represents up-regulation of genes while green represents down-regulation. Fold change values are shown in a log_2_-based format.

Hassan et al. [35] investigated the effect of a *gacA* mutation on the transcriptome of *P. fluorescens* Pf-5 and found that many genes involved in iron homeostasis were negatively regulated by the GacS/GacA two-component regulatory system. Accordingly, there is a significant degree of overlap between the transcriptomic responses of Pf-5 to *gacA* deletion and to iron limitation (Figure 3). For example, approximately half (43 of 84) of the genes that were up-regulated by more than 2-fold in response to *gacA* deletion, were also significantly up-regulated by 2-fold or more under iron-limited conditions (Table S1). These loci include 14 genes encoding ECF sigma factors, the *fagA-fumC-orfX-sodA* operon (PFL_0906-09), *pchR* (PFL_3497), the pyoverdine biosynthesis cluster, the *exbB-exbD-tonB* cluster (PFL_6067-69), and genes encoding a bacterioferritin-associated ferredoxin (PFL_4858) and haem oxygenase HemO (PFL_4628) (Table S1). Conversely, approximately one third (43 of 122) of the genes positively regulated by GacA by more than 2-fold and were significantly down-regulated by at least 2-fold under iron-limited conditions (Table S1). These loci include the hydrogen cyanide synthase genes (PFL_2577-79), cytochrome *c* oxidase *cbb3*-type gene cluster (PFL_1922-25), iron-based superoxide dismutase (PFL_4826) and flagellar-related regulators (PFL_1666-67) (Table S1). The overlapping *gacA* and iron regulons signifies that some genes involved in iron homeostasis are controlled by both iron and the GacS/GacA signal transduction cascade. It is possible that the Fur regulator interacts with a component of the GacS/GacA signal transduction cascade, although this needs to be experimentally demonstrated. A recent study [82] showed that both Fur and iron concentration affect *gacA* gene expression in *P. syringae*, but we did not observe a significant influence of iron on transcription of *gacA* in this study.

While the mechanism remains uncharacterized, a regulatory architecture linking iron and the GacA/GasS cascade might provide fine-tuning of iron homeostasis in conjunction with other environmental cues.

### Overview of Proteomic Analyses and Comparison with Transcriptional Profile

**Figure 4:**
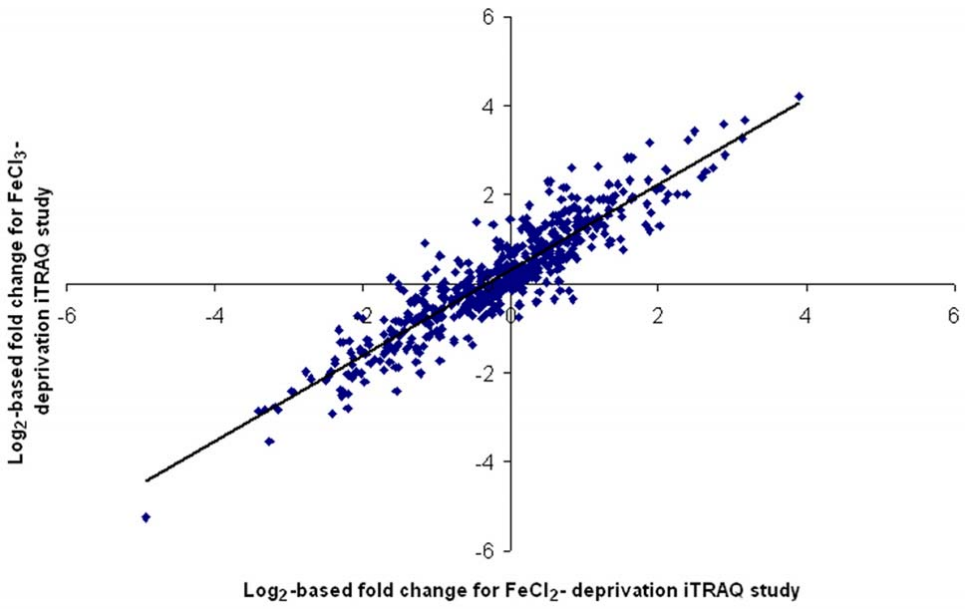
Correlation of log_2_-based fold changes of protein expression between the FeCl_2_ and FeCl_3_ iTRAQ studies. Only proteins with two or more peptides detected in the MS/MS spectra were taken into consideration. A Pearson correlation coefficient of 0.91 was observed for this comparison.

A shotgun proteomic analysis was conducted to complement our transcriptomic study and extend the results to an analysis of protein expression. The culture conditions used were identical to those used in the microarray studies. A total of 547 proteins were detected in the experiment comparing iron deprivation with either FeCl_2_ or FeCl_3_ supplementation. Of these, 168 were significantly up-regulated and 132 proteins were down-regulated in the iron-limited compared to the cultures supplemented with FeCl_2_ (Table S5). Likewise, in the experiment comparing iron deprivation to FeCl_3_ supplementation, 184 proteins were significantly up-regulated and 129 were significantly down-regulated under iron limitation (Table S5). In both the FeCl_2_ and FeCl_3_-based experiments, a greater proportion of proteins defined as being in the “protein synthesis” role category were down-regulated (Figure S4). The correlation of differentially expressed proteins detected in the FeCl_2_ and FeCl_3_ experiments was strong (Pearson correlation coefficient=0.91) (Figure 4). Overall, an under-representation of membrane-associated proteins (estimated 3.8%), as determined from the GRAVY scores, probable transmembrane domains and predicted localizations of the detected proteins, was observed in our proteomic experiment (compared to estimated 28% of the whole predicted proteome of Pf-5). Other proteomic studies utilizing iTRAQ have also observed an apparent under-representation of membrane-associated proteins [83], [84], which could be the result of inherent difficulties in membrane protein solubilization and digestion by protease [85].

**Figure 5:**
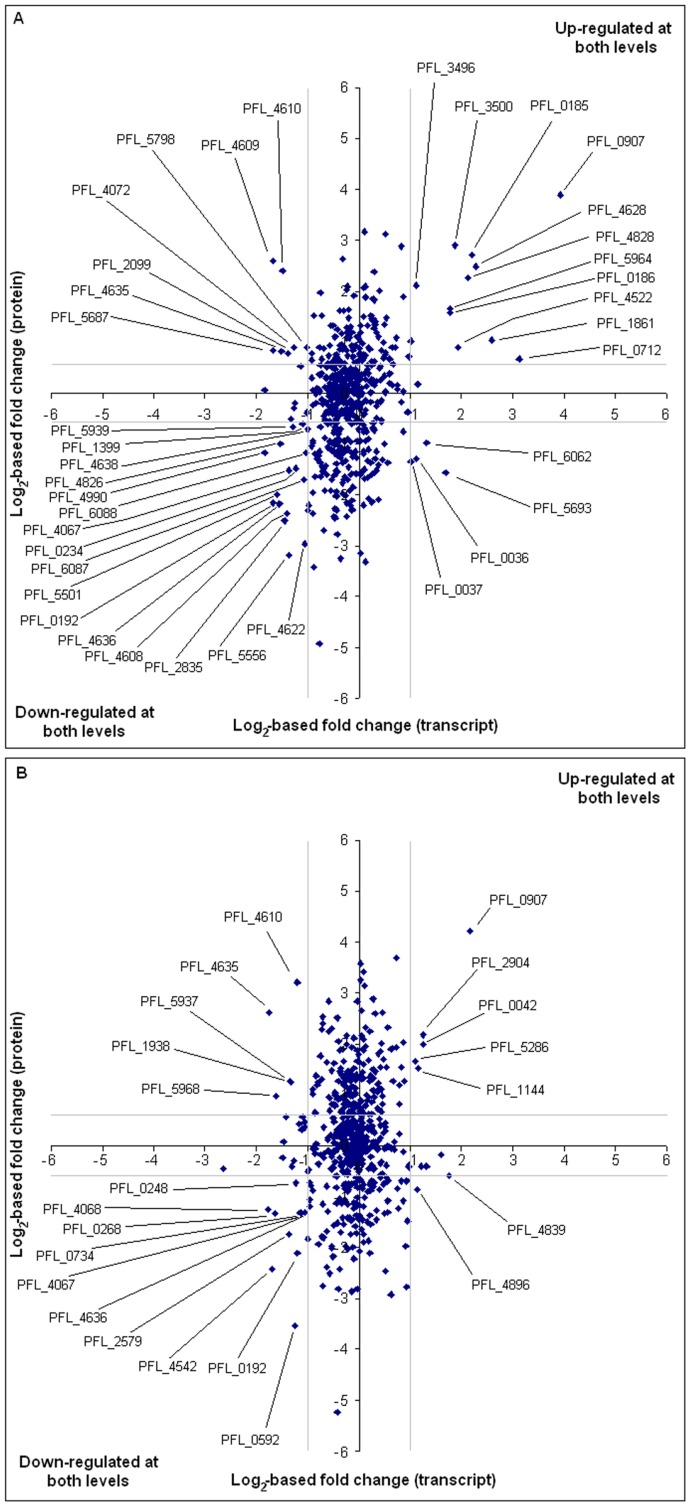
Scatterplot of fold changes of transcript versus protein expression. The charts show log_2_-based fold changes of transcript data from microarray experiments versus protein expression data derived from iTRAQ experiment for studies utilizing (A) FeCl_2_ and (B) FeCl_3_. Only proteins with at least two peptides detected in the MS/MS spectra were considered. Grey lines indicate the significance threshold. Correlations observed were modest for both comparisons with Pearson correlation coefficients of 0.31 and 0.12 respectively.

A moderate correlation between the transcriptomic and proteomic data was observed (Figure 5). Of the 385 proteins differentially expressed by Pf-5 in iron-limited versus iron-replete media (either the FeCl_2_ or FeCl_3_ form), 70 were also significantly regulated at the transcriptional level. Of these 70 proteins, 16 showed opposite trends in regulation at the transcriptional and translational levels (Figure 5; Table S5). These patterns are likely to be related to the complexity of regulation and are commonly observed in studies comparing transcript and protein abundance of bacteria under a range of experimental conditions [86], [87], [88], [89], [90]. For example, in *Leptospira interrogans*, only 25% of the outer membrane proteins that were regulated significantly by temperature were correspondingly regulated at the transcriptional level [90]. Post-transcriptional regulation involving translational efficiency as well as mRNA and protein stability is particularly important in the regulation of iron homeostasis. In *P. aeruginosa*, the Fur-controlled regulatory RNAs *prrF1* and *prrF2*, which have homologues in Pf-5 [91], mediate the translation of mRNAs encoding iron-containing proteins [92]. Additionally, regulatory RNA sequence of iron-responsive-like elements, which might confer post-transcriptional regulation by iron, have been detected in bacterial mRNA [93], [94]. Many of the 70 genes regulated only at the translational level in this study have annotated functions in primary metabolism, such as amino acid and nucleotide synthesis. For example, the proteomic analysis identified 42 ribosomal proteins to be less abundant in the iron-limited versus iron-supplemented medium, which correlates with the lower growth rate of Pf-5 observed under iron-limited conditions.

The differences in expression levels of detected proteins between FeCl_2_ and FeCl_3_-supplemented cultures were not as extensive as those observed at transcriptional level. In both experiments (FeCl_2_ and FelCl_3_), proteins involved in iron homeostasis, including pyoverdine and enantio-pyochelin biosynthesis and transport, were up-regulated in the iron-limited medium. A number of highly-expressed proteins (i.e. PFL_4088, PFL_4089, PFL_4092 and PFL_4179) involved in pyoverdine biosynthesis were not over-expressed significantly at the transcriptional level, suggesting that they are subject to post-transcriptional control (Table 5; Table S5). Two proteins encoded by genes (PFL_3496 and PFL_3500) in the enantio-pyochelin cluster were over-expressed in the iron-limited medium (Table 5). In addition to the siderophore biosynthesis proteins, components of the alternative haem uptake (Phu) system (PFL_4628 and PFL_5266) and a homologue (PFL_5964) of the HitABC iron(III) uptake system were also over-expressed under iron limitation (Table 5). Notably, the Fur protein (PFL_0824) was over-expressed by Pf-5 grown under iron-limited conditions, a phenomenon that was observed previously in *Neisseria meningitidis* [95]. It is intriguing that Fur was over-expressed in a condition where it is unlikely to perform its role as an iron-dependent repressor. Over-expression of Fur under iron-limited conditions may serve to maintain Fur abundance above a minimum threshold level in order to facilitate a more effective regulatory control [96]. Besides that, it was reported that Fur in its apo form can still function actively in regulation [97].

**Table 5:** Iron regulation of selected genes and proteins.

		Fold change (log_2_)			
Genes	Annotated functions	Iron-deprived (-FeCl_2_)		Iron-deprived (-FeCl_3_)	
		Transcript	Protein	Transcript	Protein
PFL_0613	bacterioferritin family protein	NS	**−0.76**	NS	**−1.33**
PFL_0824	ferric uptake regulation protein Fur	NS	**0.89**	NS	**1.27**
PFL_0907	fumarate hydratase, FumC_1	**3.93**	**3.89**	2.16#	**4.22**
PFL_1629	flagellin FliC	NS	−0.26	NS	**−0.69**
PFL_2090	chitin-binding protein	NS	**1.52**	NS	**2.09**
PFL_2091	chitinase ChiC	NS	**1.97**	NS	**2.11**
PFL_2579	hydrogen cyanide synthase HcnC	NS	**−1.63**	**−1.36**	**−1.75**
PFL_3496	salicyl-AMP ligase PchD	**1.11**	**2.12**	NS	**2.02**
PFL_3500	FeCT family iron-chelate ABC transporter periplasmic iron-chelate-binding protein	**1.87**	**2.91**	NS	**2.88**
PFL_3604	tryptophan halogenase PrnA	NS	**1.84**	NS	**1.79**
PFL_4088	chromophore maturation protein PvdN	NS	**2.88**	NS	**3.57**
PFL_4089	chromophore maturation protein PvdO	NS	**1.36**	NS	**1.82**
PFL_4092	TonB-dependent outermembrane ferripyoverdine receptor FpvA	NS	**1.88**	NS	**1.18**
PFL_4179	diaminobutyrate–2-oxoglutarate aminotransferase PvdH	NS	**0.90**	NS	**1.38**
PFL_4328	fumarate hydratase FumC_3	NS	**1.47**	NS	**1.00**
PFL_4628	heme oxygenase HemO	**2.28**	**2.49**	NS	**3.41**
PFL_4826	superoxide dismutase (Fe) SodB	**−1.53**	**−1.01**	**−1.13**	0.32
PFL_5266	hemin ABC transporter periplasmic hemin-binding protein PhuT	**1.01**	**1.02**	NS	**1.22**
PFL_5556	catalase KatA	**−1.37**	**−3.19**	NS	**−2.75**
PFL_5964	FeT family ferric iron ABC transporter periplasmic ferric iron-binding protein	**1.78**	**1.65**	NS	**1.94**

NS means not significant when analyzed with SAM at FDR <1% in this study. Numbers in bold denote fold changes that surpass the significant differential expression threshold defined in this study, i.e. equal to or exceeding 2-fold and 1.5-fold changes for transcript and protein levels respectively.

# Transcript level of these gene differed significantly between cells grown in iron-limited versus iron-amended media when analyzed with SAM at FDR <5%.

The abundance of several proteins involved in the oxidative stress response of Pf-5 was also altered by iron levels. The iron-independent fumarate hydratase (PFL_0907) [98] was over-expressed under iron-limited conditions while catalase KatA (PFL_5556) and iron-dependent superoxide dismutase (PFL_4826) were under-expressed at both the transcriptional and protein levels (Table 5). Another probable iron-independent fumarate hydratase (PFL_4328), which was not regulated by iron at the transcriptional level, was over-expressed at the protein level under iron-limited conditions (Table 5). Interestingly, a predicted bacterioferritin (PFL_0613, a homologue of PA4880 of *P. aeruginosa*) was under-expressed in the iron-limited medium but was not significantly regulated by iron at the transcript level (Table 5). This observation is consistent with the known post-transcriptional regulation of PA4880 by PrrF1 and PrrF2 in *P. aeruginosa* [92].

We also observed repression of the hydrogen cyanide biosynthetic protein PFL_2579, supporting similar findings from a previous study [81]. Interestingly, other proteins contributing to fungal antagonism by Pf-5, including a pyrrolnitrin biosynthetic protein (PFL_3604), a chitinase (PFL_2091), and a chitin binding protein (PFL_2090) were up-regulated under iron-limiting conditions. In line with the phenotypic observation of reduced motility of Pf-5 under low iron condition, our proteomic experiments detected decreased abundance of a flagellin protein (PFL_1629) from cultures grown in iron-limited media versus iron-replete media (Table 5).

### Effects of Iron Limitation on Expression of Proteins Containing Iron-sulfur Cluster Binding Motifs and the Machinery Involved in Iron-sulfur Cluster Biogenesis

Iron-sulfur clusters play a very important role in the activity of many enzymes in bacteria [99]. Under iron limitation, a number of Pf-5 genes encoding proteins with iron-sulfur clusters displayed decreased transcription (Table 6). In addition, many genes encoding proteins containing iron-sulfur cluster binding motifs were not affected at a transcriptional level, but their corresponding protein abundance was lower in cells grown under iron-limitation (Table 6), suggesting post-transcriptional control. A similar pattern of iron-regulated expression of iron-sulfur proteins was seen in *Yersinia pestis* [100], and also in *E. coli* where the regulatory RNA RyhB controls the expression of proteins containing iron-sulfur clusters [101]. Another possible explanation for the lower abundance of proteins containing iron-sulfur clusters, but not their corresponding transcripts, under iron-limited conditions is that the iron-sulfur cluster itself might protect the proteins against intracellular protease degradation [102].

**Table 6:** Iron regulation of selected genes encoding proteins with iron-sulfur cluster binding motifs or involved in iron-sulfur cluster assembly.

		Fold change (log_2_)			
Genes	Annotated functions	Iron-deprived (-FeCl_2_)		Iron-deprived (-FeCl_3_)	
		Transcript	Protein	Transcript	Protein
PFL_0330	formate dehydrogenase subunit beta FdsB	NS	**−1.85**	−0.62	**−1.68**
PFL_1208	ferredoxin FdxA	NS	**−1.97**	NS	**−2.21**
PFL_1241	ferredoxin-NADP reductase Fpr_1	−0.65	**−1.44**	NS	**−0.88**
PFL_1666	flagellar synthesis regulator FleN	NS	ND	**−1.55**	ND
PFL_1912	oxygen-independent coproporphyrinogen III oxidase HemN	**−1.23**	ND	**−1.52**	ND
PFL_1917	cytochrome c oxidase accessory protein CcoG	**−1.58**	ND	−0.72	ND
PFL_1929	aconitate hydratase 1 AcnA	**−1.44**	ND	−0.95	ND
PFL_2063	isopropylmalate isomerase large subunit LeuC	NS	**−0.68**	NS	**−0.69**
PFL_2578	hydrogen cyanide synthase HcnB	**−1.26**	−0.29	**−1.49**	−0.23
PFL_2633	bifunctional aconitate hydratase 2/2-methylisocitrate dehydratase AcnB	0.29	**−1.57**	NS	**−0.86**
PFL_2835	sulfite reductase (NADPH) hemoprotein subunit beta CysI_1	**−1.45**	**−2.51**	−0.51	**−2.18**
PFL_2869	cytochrome c oxidase accessory protein CcoG	**−1.04**	ND	NS	ND
PFL_2917	oxidoreductase membrane protein, FAD-binding	**−1.93**	ND	**−2.31**	ND
PFL_3902	NADH dehydrogenase subunit G NuoG	−0.75	**−1.38**	NS	**−0.67**
PFL_3904	NADH-quinone oxidoreductase, I subunit NuoI	**−1.03**	ND	NS	ND
PFL_4801	fumarate hydratase, class I	−0.76	**−4.92**	NS	**−5.23**
PFL_4858∧	bacterioferritin-associated ferredoxin, putative	NS	NA	**4.83**	ND
PFL_4964	scaffold protein IscU	0.87	**−0.76**	NS	**−1.27**
PFL_4966	iron-sulfur cluster assembly transcription factor IscR	NS	ND	**1.29**	ND
PFL_5080	ubiquinol-cytochrome c reductase, iron-sulfur subunit PetA	**−1.41**	ND	NS	ND
PFL_5693	biotin synthase BioB	**1.69**	**−1.57**	NS	**−1.87**
PFL_5877	dihydroxy-acid dehydratase IlvD	NS	**−3.14**	NS	**−2.82**

∧Pf-5 genes that contain Fur binding motifs upstream [35]. NS means not significant when analyzed with SAM at FDR <1% in this study. ND means protein is not detected from database search of the proteomic mass spectrometry data. Numbers in bold denote fold changes that surpass the significant differential expression threshold defined in this study, i.e. equal to or exceeding 2-fold and 1.5-fold changes for transcript and protein levels respectively.

Besides the effects on proteins containing iron-sulfur clusters, we also observed the consequence of iron limitation on certain genes (PFL_4964, *iscU*; and PFL_4966, *iscR*) in the well-conserved iron-sulfur cluster assembly operon *iscRSUA* (PFL_4959-66) [103]. In *E. coli*, IscR functions as a repressor for the *iscRSUA* operon when bound to an iron-sulfur cluster, which can be disrupted when the iron level is low, resulting in derepression of the operon [103]. In our study, iron limitation resulted in transcriptional up-regulation of *iscR* but down-regulation of IscU production in Pf-5 (Table 6). The apparent conflict in the influence of iron on expression of two genes in a single polycistronic transcript could be due to post-transcriptional regulation of IscU. In *E. coli*, the post-transcriptional regulation of the *iscRSUA* polycistronic transcript is mediated by RyhB [101] through partial degradation of the transcript, leaving only the transcript segment encoding IscR intact and capable of protein expression [104]. It is yet to be ascertained if *P. fluorescens* Pf-5 employs similar post-transcriptional regulatory control of its *iscRSUA* operon.

### Conclusions

In this study, we examined the transcriptomic and proteomic impact of iron limitation on the biocontrol bacterium *P. fluorescens* Pf-5 by evaluating iron-limited cultures in comparison to both FeCl_2_- and FeCl_3_- amended cultures. The effects of both forms of iron were very similar despite some variation in levels of transcriptional regulation for some genes. Under iron limitation, we observed changes in transcription of genes encoding iron homeostasis functions such as siderophore biosynthesis and other iron uptake systems. Based on the transcriptional data, it seems likely that, of the five *exbB-exbD-tonB* transport clusters found in Pf-5, the PFL_6067-69 gene cluster encodes proteins facilitating energy transduction of various TonB-dependent receptors expressed under iron limitation. Iron limitation resulted in a number of unexpected responses, particularly the increased transcription of the ribosomal protein L36 gene as well as reduced flagellar biosynthesis gene expression and motility. Interestingly, certain genes or proteins involved in biosynthesis of secondary metabolites such as 2,4-diacetylphloroglucinol (DAPG), orfamide A and pyrrolnitrin, as well as the *reb*-homologous gene cluster and a chitinase were over-expressed under low iron conditions. The phenomena of increased production of bioactive products by bacteria under low iron conditions have been observed, such as exotoxin A in *P. aeruginosa* [105] and microcystin-LR in *Microcystis aeruginosa* [106]. The proteomic data from iTRAQ also revealed differences in the abundance of proteins involved in iron homeostasis. Importantly, comparison of the transcriptomic data with the proteomic data revealed a number of candidate proteins that might be post-transcriptionally regulated, especially those proteins that harbour iron-sulfur cluster binding motifs.

## Materials and Methods

### Bacterial Strains and Growth Conditions

For microarray and proteomic analyses, *Pseudomonas fluorescens* Pf-5 was grown in M9 minimal media supplemented with 100 µM calcium chloride (CaCl_2_), 2 mM magnesium sulphate (MgSO_4_) and 0.4% glucose [107]. The iron-replete cultures were supplemented with either 100 µM iron(II) chloride (FeCl_2_) or 100 µM iron(III) chloride (FeCl_3_), while iron-limited cultures were not supplemented with iron in any form. Both iron stock solutions were prepared by dissolving the iron chloride crystals in deionized water. The cultures were grown aerobically with shaking at room temperature to late exponential phase; optical densities (at 600 nm wavelength) of harvested cultures were approximately 0.7 for iron-limited cultures and 1.1 for iron-amended cultures (Figure S1). The pH of iron-limited and iron-supplemented culture media was tested and found to be close to neutral.

### RNA Extraction

Extraction of RNA was performed using the PureLink Micro-to-Midi Total RNA Purification System (Invitrogen) with an initial extraction using Trizol reagent (Invitrogen) as per the manufacturer’s instructions. The concentration and purity of mRNA extracted was determined spectrophotometrically using a Nanodrop ND1000 spectrophotometer (NanoDrop Technologies).

### cDNA Synthesis and Labeling

Reverse transcription of RNA samples was performed using the SuperScript Plus Indirect cDNA Labeling System (Invitrogen) with random hexamer primers. Approximately 7 µg of total RNA was used for cDNA synthesis in each reaction. The cDNA samples were labeled with either Alexa Fluor 555 or Alexa Fluor 647 (Invitrogen).

### Microarray Experiments

The quality of labeled cDNA was confirmed spectrophotometrically using a Nanodrop ND1000 spectrophotometer (NanoDrop Technologies), prior to hybridization. Microarray slides spotted with 70-mer DNA oligonucleotides representing almost all of the open reading frames annotated in the genome of Pf-5 [35]. Each microarray experiment consisted of at least three biological replicates and three technical replicates. Flip-dye experiments were also performed to ensure a lack of dye bias in the results. The hybridization procedure was performed as described previously [108]. Hybridized slides were scanned with an Axon 4000B scanner with GenePix 4.0 software. The microarray data was analyzed with Spotfinder (TIGR) and normalized using the LOWESS algorithm in the TIGR-MIDAS package with block mode and a smoothing parameter value of 0.33. Genes significantly regulated by iron were identified using the Statistical Analysis of Microarrays (SAM) algorithm with a false discovery rate (FDR) of less than 1% [109]. Comparisons between transcriptional profiles were performed with MultiExperiment Viewer (MeV) 4.1 software [110]. A fold change of at least 2 was considered to be significantly differentially expressed. The microarray datasets have been deposited in the Gene Expression Omnibus (GEO) database (http://www.ncbi.nlm.nih.gov/geo/) with the accession number GSE29322. The details provided for the microarray datasets are in compliance with the MIAME guideline [111].

### qRT-PCR Validation of Microarray

Quantitative reverse-transcriptase polymerase chain reaction (qRT-PCR) analyses were conducted on a selection of differentially expressed genes to validate the microarray results. For each sample, 2.5 µg of RNA was reverse-transcribed to cDNA with the SuperScript VILO cDNA Synthesis Kit (Invitrogen) according to the manufacturer’s instructions. The resulting cDNA samples were used as template for 10 µl real-time PCR reactions containing EXPRESS SYBR GreenER qPCR SuperMix with Premixed ROX (Invitrogen) and gene-specific primers. The primers were designed using Primer3 software [112] and tested for their amplification efficiencies. Genes encoding ribosomal protein S7 and S12 (PFL_5586 and PFL_5587, respectively) were utilized as internal references. The reactions were performed in a Mastercycler ep Realplex^4^ S (Eppendorf). At least three replicates were performed for each sample. Cycle threshold (C_T_) values were determined using Eppendorf Mastercycler ep Realplex 2.2 software and the ΔΔC_T_ method was used to calculate the relative template abundance in control versus experimental samples [113].

### Motility Assay

Motility tests were performed to determine the effect of iron-limited conditions on swarming phenotypes. Tests were performed on 0.6% agar-containing M9 minimal media plate supplemented with 100 µM CaCl_2_, 2 mM MgSO_4_ and 0.4% glucose. Varying concentrations of FeCl_2_, from 0 to 100 µM, were added into the agar plates. Overnight cultures of Pf-5 were diluted to OD_600_=0.2, 2 µl was placed on the center of the agar surface, and plates were incubated at 25°C for two days. A *gacA* mutant (JL4577) [35] served as a swarming motility negative control.

### iTRAQ Labeling of Protein Samples

Cultures of *P. fluorescens* Pf-5 cultures were grown in M9 broth medium as described above. The bacterial pellets from cultures were washed with phosphate-buffered saline solution (pH 7.4) before being lysed in lysis buffer [20 mM HEPES, 150 mM sodium chloride (NaCl), DNase I (Sigma-Aldrich), and protease inhibitor cocktail (Sigma-Aldrich)] by mechanical lysis in a FastPrep FP120 bead beater (BIO101/Savant, Q-Biogene) at 5.5 Throw for 30 seconds. The concentrations of the harvested protein extracts were determined using Bradford assays (Bio-Rad) and the quality examined with SDS-PAGE. An 8-plex iTRAQ (isobaric tag for relative and absolute quantitation) system (Applied Biosystems) was used. The protein samples were reduced and alkylated with 100 mM tris-2-carboxyethylphosphine (TCEP) and 200 mM methyl methane thiosulfonate (MMTS) and proteolysed with trypsin for 16 hours at 37°C. The samples were labeled with iTRAQ tags (Applied Biosystems) according to the manufacturer’s protocol. Three biological replicates of iron-limited cells (labeled with iTRAQ 113, 116 and 119) and FeCl_3_ supplemented cells (labeled with iTRAQ 115, 118 and 121) were used, while two biological replicates supplemented with FeCl_2_ (labeled with iTRAQ 114 and 117) were used. The samples were grown in three batches where two batches consisted of iron-limited, FeCl_2_-supplemented and FeCl_3_-supplemented cultures while the last batch consisted only of iron-limited and FeCl_3_-supplemented cultures (constrained due to limited number of iTRAQ labels available).

### Strong Cation Exchange Fractionation

Strong cation exchange chromatography was performed in an Agilent 1100 quaternary HPLC pump (Agilent Technologies) with a PolyC PolySulfoethyl A 200 mm×2.1 mm 1.5 µm 200A column. The buffers used were buffer A [5 mM phosphate, 25% acetonitrile (CH_3_CN), pH 2.7] and buffer B [5 mM phosphate, 350 mM potassium chloride (KCl), 25% CH_3_CN, pH 2.7]. Labeled samples were suspended in buffer A and loaded into the column. The peptides were separated as the buffer B concentration was increased from 10% to 45% over a period of 70 minutes. Subsequently, the buffer B concentration was quickly increased to 100% for 10 minutes at a flow rate of 300 µl/min. The fractions collected were dried in a SpeedVac prior to analysis with nanoLC electrospray (MS/MS).

### Mass Spectrometry

NanoLC electrospray (MS/MS) was performed using the Agilent 1100 nanoLC system (Agilent Technologies) and QStar Elite MS/MS system (Applied Biosystems). The samples were resuspended in 100 µl of loading/desalting solution [0.1% trifluoroacetic acid (TFA), 2% CH_3_CN and 97.9% H_2_O]. 40 µl of each sample was loaded into a reverse phase peptide Captrap (Michrom BioResources) and then desalted with the desalting solution at 10 µl per minute for 15 minutes. After this procedure, the trap was switched on line with a ProteCol column (150 µm×10 cm C18 3 µm 300A) (SGE GmbH). In Channel 1, the buffer consisted of 97.9% H_2_O, 0.1% formic acid (HCO_2_H), 2% CH_3_CN. Channels 2A and 2B contained 99.9% H_2_O, 0.1% HCO_2_H and 90% CH_3_CN, 9.9% H_2_O, 0.1% HCO_2_H, respectively. To elute the peptides from the column, the concentration of Channel 2B was changed from 5% to 100% at 500 nL per minute for a period of 93 minutes in three linear gradient steps. After peptide elution, the column was flushed with Channel 2B buffer and equilibrated with 95% Channel 2A buffer for 8 minutes before the next sample was injected. Peptides eluted from the reverse phase nanoLC were analyzed in an information-dependant acquisition mode (IDA) with positive ion nanoflow electrospray. A TOFMS survey scan was performed (m/z 380–1600, 0.5 second) in the IDA mode. The three most intense multiply charged ions (counts >70) were put into MS/MS analysis sequentially. A modified Enhanced All Q2 transition setting was utilized, which favours low mass ion detection so that iTRAQ label intensity signals were enhanced for quantification. The MS/MS spectra (mass range m/z 100–1600) were collected for 2 seconds.

### Proteomic Data Analysis

ProteinPilot 4.0 software (Applied Biosystems) was used to analyze the MS/MS data generated. The amino acid sequence database for *P. fluorescens* Pf-5 was obtained from the NCBInr database. A thorough ID search was performed using the Paragon method with Biological modifications selected in ID Focus, False Discovery Rate analysis enabled and Background Correction setting used. The iTRAQ reagent correction factors were entered into the iTRAQ Isotope Correction Factors table. For quantification purpose, the software will select peptides that meet its default set of criteria, such as having peptide ID confidence of at least 15.0%, iTRAQ modification and a good signal-to-noise ratio. The results of this analysis were filtered to include only proteins with at least 2 peptides detected in the MS/MS spectra with 95% confidence and having Unused ProtScore >2, which is the measure of the protein identification confidence where a score of 2 corresponds to a 99% confidence limit. Protein abundance ratios from comparison of iron-limited culture against iron-supplemented cultures from each sampling batch were calculated. The ratios for each category (i.e. either iron-limited versus FeCl_2_-supplemented cultures or iron-limited versus FeCl_3_-supplemented cultures) from all the batches were subsequently averaged and reported. A fold change of at least 1.5 was considered significant [89].

### Bioinformatics Analysis

Useful information pertaining to iron-sulfur cluster binding motifs and predicted operons were garnered from Pseudomonas Genome Database [114] and EcoCyc [115]. Membrane association characteristics of the detected proteins from the proteomic experiment were evaluated as follows: hydrophobic properties were calculated as grand average of hydropathy (GRAVY) scores [116] with Sequence Manipulation Suite [117], transmembrane spanning domains were examined using TMHMM v2.0 [118], and predicted localization of proteins was inferred using PSORTb v3.0.2 [119]. Conversions of identifiers between databases were facilitated by db2db software in bioDBnet [120].

## Supporting Information

Figure S1 **Growth curves of** ***P. fluorescens*** **Pf-5 in iron-limited minimal medium and in minimal medium amended with either FeCl_2_ or FeCl_3_.**
(DOC)

Figure S2 **Role categories of genes (as defined by Hassan et al.** [**35**] **) of microarray data.**
(DOC)

Figure S3 **Validation of microarray data.**
(DOC)

Figure S4 **Role categories of genes (as defined by Hassan et al.** [**35**] **) analysis of iTRAQ proteomic data.**
(DOC)

Table S1 **Iron-regulated genes identified by SAM analysis using a false discovery rate (FDR) of less than 1% and at least a 2-fold change in transcript level.**
(DOC)

Table S2 **Primer sequences for qRT-PCR validation of microarray data.**
(DOC)

Table S3 **Regulation of genes with predicted upstream Fur binding sites.**
(DOC)

Table S4 **Regulation of clusters containing genes encoding sigma factors, TonB-dependent receptors, and anti-sigma factor regulators as determined by SAM analysis.**
(DOC)

Table S5 **Comparison of proteomic data from iTRAQ experiments versus transcriptomic data from microarray experiments.**
(XLS)
