# Supplementary material for: The Effect of Iron Limitation on the Transcriptome and Proteome of Pseudomonas fluorescens Pf-5
Source: PLoS One. 2012 Jun 18;7(6):e39139. doi: 10.1371/journal.pone.0039139 (PMC3377617; doi:10.1371/journal.pone.0039139)
Supplement: Figure S1 — Growth curves of P. fluorescens Pf-5 in iron-limited minimal medium and in minimal medium amended with either FeCl2 or FeCl3. (DOC) [file pone.0039139.s001.doc]

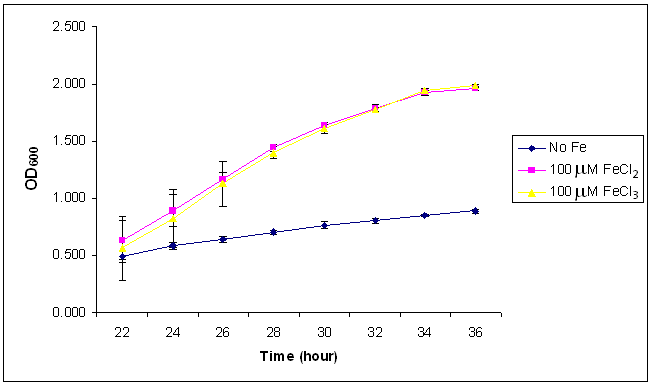


Figure S1. Growth curves of *P. fluorescens* Pf-5 in iron-limited minimal medium and in minimal medium amended with either FeCl_2_ or FeCl_3_. The optical densities at 600 nm wavelength were taken from 22^nd^ hour to 36^th^ hour at intervals of 2 hours. Measurements were performed on triplicate samples.
