# Supplementary material for: The Effect of Iron Limitation on the Transcriptome and Proteome of Pseudomonas fluorescens Pf-5
Source: PLoS One. 2012 Jun 18;7(6):e39139. doi: 10.1371/journal.pone.0039139 (PMC3377617; doi:10.1371/journal.pone.0039139)
Supplement: Figure S2 — Role categories of genes (as defined by Hassan et al. [35] ) of microarray data. (DOC) [file pone.0039139.s002.doc]

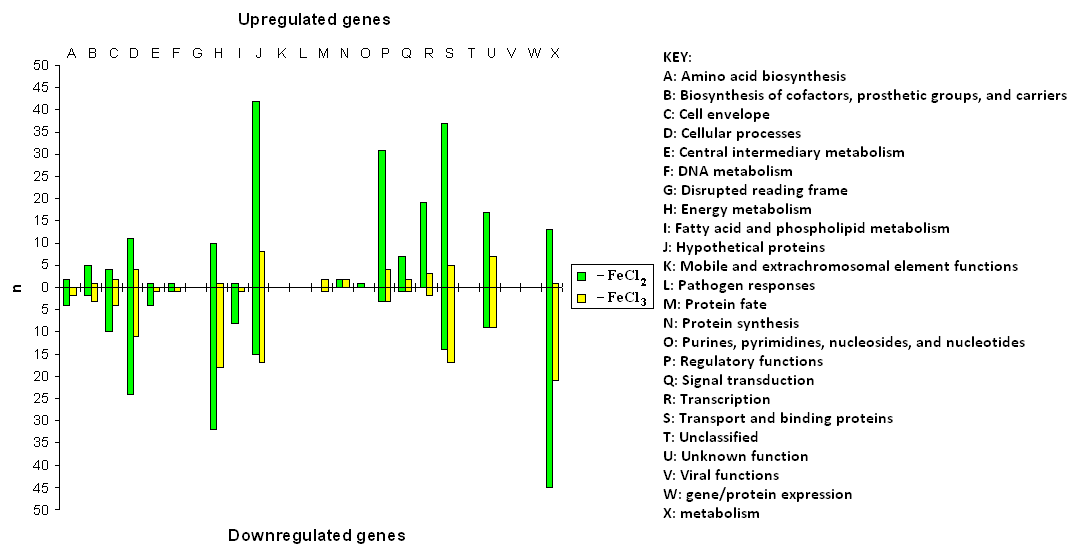


Figure S2. Role categories of genes (as defined by Hassan et al. [35]) of microarray data. The number of genes that are up-regulated and down-regulated in Pf-5 grown in an iron-limited medium versus the medium amended with FeCl_2_ (Green) or FeCl_3_ (Yellow) are categorized according to the role categories. Some genes can be in more than one category and so may be counted more than once.
