# Supplementary material for: The Effect of Iron Limitation on the Transcriptome and Proteome of Pseudomonas fluorescens Pf-5
Source: PLoS One. 2012 Jun 18;7(6):e39139. doi: 10.1371/journal.pone.0039139 (PMC3377617; doi:10.1371/journal.pone.0039139)
Supplement: Figure S3 — Validation of microarray data. (DOC) [file pone.0039139.s003.doc]

A.

B.

Figure S3. Validation of microarray data. Correlation analysis of log_2_-based fold changes between microarray data and qRT-PCR data for 22 genes of *P. fluorescens* Pf-5. Charts depict plots of microarray log_2_-based fold changes versus qRT-PCR log_2_-based fold changes for transcripts of genes in cultures of Pf-5 grown in iron-limited medium versus (A) a FeCl_2_ amended medium (B) a FeCl_3_ amended medium. A Pearson correlation coefficient of 0.86 and correlation of coefficient (R^2^) of 0.74 was observed for (A) and a Pearson correlation coefficient of 0.93 and correlation of coefficient (R^2^) of 0.86 was observed for (B).
