# Supplementary material for: The Effect of Iron Limitation on the Transcriptome and Proteome of Pseudomonas fluorescens Pf-5
Source: PLoS One. 2012 Jun 18;7(6):e39139. doi: 10.1371/journal.pone.0039139 (PMC3377617; doi:10.1371/journal.pone.0039139)
Supplement: Figure S4 — Role categories of genes (as defined by Hassan et al. [35] ) analysis of iTRAQ proteomic data. (DOC) [file pone.0039139.s004.doc]

**
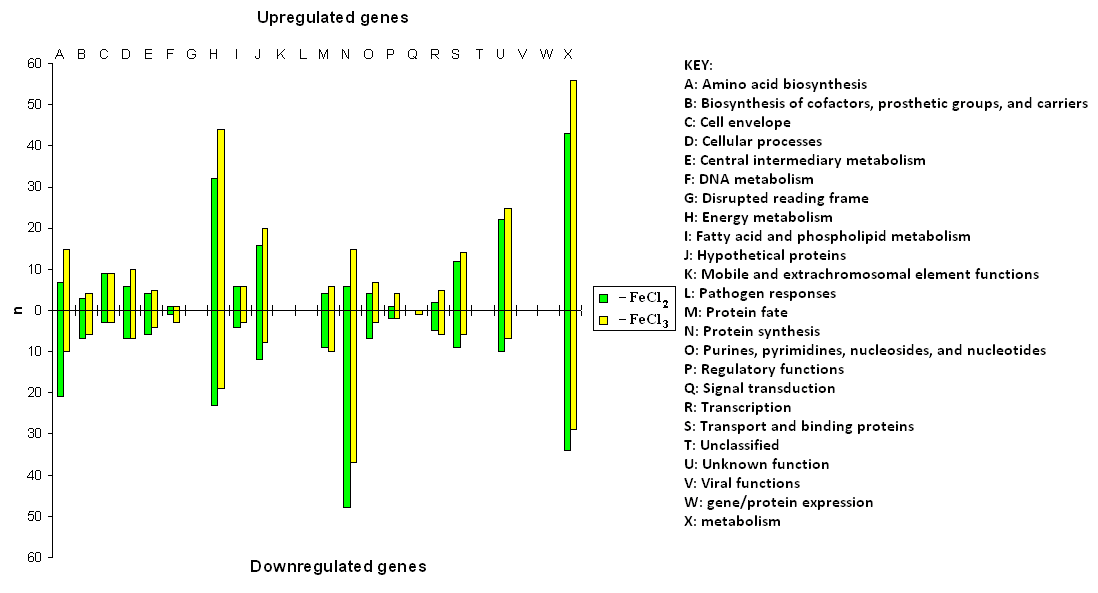
**

Figure S4. Role categories of genes (as defined by Hassan et al. [35]) analysis of iTRAQ proteomic data. The number of proteins that are up-regulated and down-regulated in Pf-5 grown in an iron-limited medium versus the medium amended with FeCl_2_ (Green) or FeCl_3_ (Yellow) are categorized according to the role categories. Some predicted gene products are in more than one category and so may be counted more than once.
