# Supplementary material for: The Effect of Iron Limitation on the Transcriptome and Proteome of Pseudomonas fluorescens Pf-5
Source: PLoS One. 2012 Jun 18;7(6):e39139. doi: 10.1371/journal.pone.0039139 (PMC3377617; doi:10.1371/journal.pone.0039139)
Supplement: Table S1 — Iron-regulated genes identified by SAM analysis using a false discovery rate (FDR) of less than 1% and at least a 2-fold change in transcript level. (DOC) [file pone.0039139.s005.doc]

Table S1. Iron-regulated genes identified by SAM analysis using a false discovery rate (FDR) of less than 1% and at least a 2-fold change in transcript level. Certain genes having smaller (i.e., < 2-fold) transcriptional responses to iron are included for comparative purposes. Fold changes are reported in log_2_-based format. Results were tabulated in (A) for genes unregulated in an iron-limited culture medium and (B) for genes down-regulated in an iron-limited medium.

(A) Genes whose transcripts were up-regulated under iron limitation conditions and comparison with Pf-5 *gacA* mutant strain study [35], *P. aeruginosa* PAO1 iron limitation studies (performed by Ochsner

et al. [27] and Palma et al. [28]), and *P. syringae* DC3000 iron limitation study (performed by Bronstein et al. [29]). – FeCl_2_ and – FeCl_3_ respectively represent FeCl_2_ and FeCl_3_ deprivation studies.

| Gene ID | Annotated function | – FeCl_2_ | – FeCl_3_ | *gacA* mutant [35] | PAO1 ortho-logues | Ochsner  et al. [27] | Palma  et al. [28] | DC3000 orthologues | Bronstein  et al. [29] |
| --- | --- | --- | --- | --- | --- | --- | --- | --- | --- |
| PFL_0036 | tryptophan synthase, alpha subunit TrpA | 1.12 | NS | NS | PA0035 | NS | NS | PSPTO0159 | NS |
| PFL_0037 | tryptophan synthase, beta subunit TrpB | 1.02 | NS | NS | PA0036 | NS | NS | PSPTO0158 | NS |
| PFL_0042 | bacterial luciferase family protein | NS | 1.25 | NS | PA2483 | NS | NS | PSPTO0153 | NS |
| PFL_0126 | sigma factor regulatory protein, FecR/PupR family | 2.27 | 0.93 | 1.58 | PA2467 | 4.91 | 4.24 | — | — |
| PFL_0127^ | RNA polymerase sigma-70 factor, ECF subfamily | 3.12 | 0.83 | 1.68 | PA2468 | 4.91 | 4.28 | — | — |
| PFL_0145^ | RNA polymerase sigma-70 factor, ECF subfamily | 4.40 | NS | 3.19 | — | — | — | — | — |
| PFL_0146 | sigma factor regulatory protein, FecR/PupR family | 2.88 | NS | 2.17 | — | — | — | — | — |
| PFL_0151 | prolyl-tRNA synthetase | 1.43 | NS | NS | — | — | — | — | — |
| PFL_0152 | conserved hypothetical protein | 1.15 | NS | NS | — | — | — | — | — |
| PFL_0159 | hypothetical protein | 1.23 | NS | NS | — | — | — | — | — |
| PFL_0183 | RebB protein | 0.91 | 1.25 | NS | — | — | — | — | — |
| PFL_0184 | rebB protein | NS | 1.31 | -1.20 | — | — | — | — | — |
| PFL_0185 | succinate-semialdehyde dehydrogenase gabD | 2.21 | NS | NS | PA0265 | 2.32 | 1.20 | PSPTO0300 | NS |
| PFL_0186 | 4-aminobutyrate transaminase gabT | 1.77 | NS | NS | PA0266 | 3.00 | 1.00 | PSPTO0301 | NS |
| PFL_0309 | conserved hypothetical protein | 1.06 | NS | NS | — | — | — | PSPTO5392 | 4.35 |
| PFL_0354 | hypothetical protein | 1.66 | 1.81 | NS | — | — | — | — | — |
| PFL_0370 | lipoprotein | 2.42 | NS | 1.77 | — | — | — | PSPTO1409 | -2.31 |
| PFL_0538 | thiamin biosynthesis protein ThiC | 1.07 | NS | NS | PA4973 | 2.00 | NS | PSPTO4976 | NS |
| PFL_0573 | iron ABC transporter permease FbpB | 1.35 | NS | 0.49 | PA4688 | NS | 2.87 | — | — |
| PFL_0574 | iron ABC transporter substrate-binding protein FbpA | 2.08 | 1.06 | 1.26 | PA4687 | NS | 1.72 | — | — |
| PFL_0681 | hypothetical protein | 1.16 | NS | NS | — | — | — | PSPTO4846 | NS |
| PFL_0688 | TadE family protein | 1.19 | NS | NS | — | — | — | PSPTO4834 | NS |
| PFL_0700 | response regulator receiver domain-containing protein | 1.30 | NS | NS | — | — | — | PSPTO4848 | NS |
| PFL_0709 | Cu(I)-responsive transcriptional regulator CueR | 1.14 | NS | NS | PA4778 | NS | NS | PSPTO0749 | NS |
| PFL_0710 | copper-translocating P-type ATPase | 1.20 | NS | NS | PA3920 | NS | NS | PSPTO0750 | NS |
| PFL_0712 | copper chaperone CopZ, putative | 3.14 | NS | NS | — | — | — | PSPTO0752 | NS |
| PFL_0835 | ribosomal RNA large subunit methyltransferase J, RrmJ | NS | 1.06 | 0.77 | PA4752 | NS | NS | PSPTO4498 | NS |
| PFL_0865 | oxidoreductase, 2OG-Fe(II) oxygenase family | 3.03 | NS | NS | PA4515 | 5.17 | 2.32 | — | — |
| PFL_0866 | Sel1 domain protein | 2.15 | NS | NS | PA4516 | 5.17 | NS | — | — |
| PFL_0906^ | fagA protein FagA | 4.93 | 2.07 | 2.96 | PA4471 | 6.89 | 6.20 | PSPTO4462 | NS |
| PFL_0907 | fumarate hydratase, class II, FumC_1 | 3.93 | NS | 2.32 | PA4470 | 6.89 | 5.13 | PSPTO4461 | 6.94 |
| PFL_0908 | hypothetical protein | 4.60 | NS | 2.26 | PA4469 | 6.89 | 6.28 | PSPTO4460 | 7.52 |
| PFL_0909 | superoxide dismutase, Mn, SodA1 | 3.27* | 1.13* | 2.87 | PA4468 | 6.89 | 5.70 | PSPTO4459 | 6.77 |
| PFL_0910 | zinc(II)-iron(II) family metal cation transporter permease | 2.44 | NS | NS | PA4467 | 6.89 | 4.19 | — | — |
| PFL_0932 | TonB-dependent receptor | 1.49 | NS | 0.14 | PA4837 | NS | NS | PSPTO2463 | NS |
| PFL_0983 | sigma factor regulatory protein FecR | 1.72 | NS | 0.93 | PA3900 | 3.70 | 2.46 | PSPTO1208 | 2.76 |
| PFL_0984^ | RNA polymerase sigma factor FecI | 1.89 | NS | 1.32 | PA3899 | 3.70 | 3.00 | PSPTO1209 | NS |
| PFL_0985^ | hypothetical protein | 1.71 | NS | NS | — | — | — | PSPTO1210 | 1.26 |
| PFL_1045 | ornithine cyclodeaminasefamily protein | 1.57 | NS | NS | PA3862 | NS | NS | — | — |
| PFL_1048 | general L-amino acid ABC transporter, periplasmic L-amino acid-binding protein AapJ | 1.34 | NS | NS | PA3858 | NS | NS | PSPTO1255 | NS |
| PFL_1144 | OmpA family lipoprotein | NS | 1.15 | NS | PA3692 | NS | NS | PSPTO1506 | NS |
| PFL_1207 | RNA polymerase sigma factor RpoS | 1.14 | NS | NS | PA3622 | NS | NS | PSPTO1565 | NS |
| PFL_1259 | hypothetical protein | 1.06 | 0.90 | NS | PA4390 | NS | NS | PSPTO4381 | 2.61 |
| PFL_1373 | RNA polymerase sigma-70 factor, ECF subfamily | 1.68 | 0.70 | 1.81 | PA1300 | 5.52 | NS | — | — |
| PFL_1417 | TonB-dependent outer membrane receptor | 1.36 | NS | NS | — | — | — | — | — |
| PFL_1420 | hypothetical protein | 1.04 | NS | NS | — | — | — | — | — |
| PFL_1563 | hypothetical protein | NS | 1.25 | NS | — | — | — | — | — |
| PFL_1568 | transcriptional regulator, LysR family | 1.02 | NS | NS | — | — | — | — | — |
| PFL_1828 | hypothetical protein | NS | 1.41 | NS | — | — | — | — | — |
| PFL_1861 | methylisocitrate lyase PrpB | 2.60 | NS | NS | PA0796 | NS | NS | PSPTO2287 | NS |
| PFL_1862 | 2-methylcitrate synthase PrpC | 1.68 | NS | NS | PA0795 | NS | NS | PSPTO2288 | NS |
| PFL_1873 | hypothetical protein | -0.93 | 1.09 | NS | — | — | — | PSPTO2296 | NS |
| PFL_1882 | benzoate transporter family protein | 1.56 | 0.90 | NS | PA1651 | NS | NS | — | — |
| PFL_1900^ | DNA-binding protein | 1.16 | 0.97 | NS | — | — | — | — | — |
| PFL_1947 | hypothetical protein | -0.45* | 1.37* | NS | — | — | — | PSPTO3512 | NS |
| PFL_2057 | hypothetical protein | 1.03 | NS | NS | PA3132 | NS | NS | — | — |
| PFL_2060 | heat-shock protein IbpA | NS | 1.61 | 0.93 | PA3126 | NS | NS | PSPTO2170 | NS |
| PFL_2143 | transcriptional regulator, LuxR family | NS | 1.48 | NS | — | — | — | PSPTO2828 | NS |
| PFL_2147 | Nonribosomal peptide synthetase OfaC | 1.41 | NS | NS | — | — | — | — | — |
| PFL_2158 | hypothetical protein | 1.13 | NS | NS | — | — | — | — | — |
| PFL_2161 | acyl-CoA dehydrogenase family protein | 1.17 | NS | NS | PA4199 | NS | NS | — | — |
| PFL_2162 | AMP-binding domain protein | 1.48 | NS | NS | PA4198 | NS | NS | — | — |
| PFL_2216 | quinoprotein ethanol dehydrogenase PedE | 1.29 | NS | NS | PA1982 | NS | NS | — | — |
| PFL_2271 | hypothetical protein | 1.08 | NS | NS | — | — | — | — | — |
| PFL_2291^ | RNA polymerase sigma factor, FecI family | 3.27 | NS | 1.54 | PA1912 | NS | NS | PSPTO1203 | NS |
| PFL_2292 | sigma factor regulatory protein, FecR/PupR family | 1.88 | NS | 0.93 | — | — | — | — | — |
| PFL_2303 | TROVE domain protein | 2.65 | 1.69 | NS | — | — | — | — | — |
| PFL_2304 | RtcB family protein | 1.62 | NS | NS | PA4583 | NS | NS | — | — |
| PFL_2305 | PBS lyase HEAT-like repeat domain protein | 1.33 | NS | NS | — | — | — | — | — |
| PFL_2326 | transcriptional regulator, AraC family | 1.27 | NS | NS | — | — | — | — | — |
| PFL_2363^ | RNA polymerase sigma-70 factor, ECF subfamily | 2.51* | 0.83* | 0.68 | — | — | — | — | — |
| PFL_2364 | sigma factor regulatory protein, FecR/PupR family | 2.50 | NS | 1.00 | — | — | — | — | — |
| PFL_2393^ | RNA polymerase sigma-70 family protein | 1.48 | NS | 1.00 | — | — | — | — | — |
| PFL_2432 | L-2-hydroxyglutarate oxidase IhgO | 2.74 | NS | NS | — | — | — | — | — |
| PFL_2433 | transcriptional regulator, GntR family | 1.89 | NS | NS | — | — | — | — | — |
| PFL_2490^ | drug resistance transporter, EmrB/QacA subfamily | 1.06 | 0.81 | NS | PA1316 | 2.00 | 1.77 | — | — |
| PFL_2528 | sigma factor regulatory protein PupR | 1.85 | 0.46 | 0.77 | — | — | — | — | — |
| PFL_2529^ | RNA polymerase sigma factor PupI | 1.23 | 0.52 | 0.68 | — | — | — | — | — |
| PFL_2664 | sensor protein PfeS | 1.22 | NS | 0.38 | PA2687 | NS | NS | — | — |
| PFL_2665 | transcriptional activator PfeR | 1.95* | 0.64* | 1.32 | PA2686 | NS | 4.84 | — | — |
| PFL_2780 | hypothetical protein | 1.02 | NS | NS | — | — | — | — | — |
| PFL_2883 | hypothetical protein | 1.02 | NS | NS | — | — | — | — | — |
| PFL_2884 | malto-oligosyltrehalose synthase TreY | 1.19 | NS | NS | PA2162 | NS | NS | PSPTO3128 | NS |
| PFL_2885 | 4-alpha-glucanotransferase MalQ | 1.01 | NS | NS | PA2163 | NS | NS | PSPTO3127 | NS |
| PFL_2894 | hypothetical protein | 1.43 | NS | NS | — | — | — | — | — |
| PFL_2901 | hypothetical protein | 1.71 | NS | NS | PA2384 | 7.21 | 1.26 | PSPTO5638 | NS |
| PFL_2904 | hypothetical protein | NS | 1.27 | NS | — | — | — | — | — |
| PFL_2920 | hypothetical protein | 1.11 | NS | NS | — | — | — | — | — |
| PFL_2924 | hypothetical protein | 1.06 | NS | NS | — | — | — | — | — |
| PFL_2951 | periplasmic putrescine-binding protein | 1.01 | NS | NS | — | — | — | — | — |
| PFL_3030 | hypothetical protein | 1.46 | NS | NS | — | — | — | — | — |
| PFL_3031 | major facilitator superfamily protein | 1.92 | NS | NS | — | — | — | — | — |
| PFL_3095 | hypothetical protein | 1.17 | NS | NS | — | — | — | — | — |
| PFL_3099 | hypothetical protein | 1.14 | NS | NS | — | — | — | — | — |
| PFL_3156 | RNA polymerase sigma-70 factor, ECF subfamily | 1.05 | NS | 0.77 | — | — | — | — | — |
| PFL_3177 | TonB-dependent outer membrane receptor | 1.14 | NS | NS | — | — | — | — | — |
| PFL_3255 | ferrous iron permease EfeU | 2.76 | 1.25 | 1.54 | — | — | — | PSPTO3596 | 1.19 |
| PFL_3287 | hypothetical protein | 1.17 | NS | NS | PA3089 | NS | NS | PSPTO3797 | NS |
| PFL_3313^ | RNA polymerase sigma-70 factor, ECF subfamily | 1.67 | NS | 1.14 | — | — | — | — | — |
| PFL_3314 | sigma factor regulatory protein, FecR/PupR family | 1.05 | NS | 0.38 | — | — | — | — | — |
| PFL_3330 | sensory box-containing diguanylate cyclase, putative | 1.07 | NS | NS | PA2072 | NS | NS | — | — |
| PFL_3358 | transcriptional regulator, LuxR family | 1.20 | NS | NS | PA3771 | NS | NS | — | — |
| PFL_3419 | hypothetical protein | 1.21 | NS | NS | — | — | — | — | — |
| PFL_3427 | hypothetical protein | 1.09* | -0.54* | NS | — | — | — | — | — |
| PFL_3459 | acetyltransferase, GNAT family | 1.15 | NS | NS | — | — | — | — | — |
| PFL_3482^ | transcriptional regulator, MarR family/acetyl transferase, GNAT family | 1.06 | NS | NS | — | — | — | — | — |
| PFL_3483^ | RNA polymerase sigma-70 factor, ECF subfamily | 1.87 | NS | 1.32 | — | — | — | — | — |
| PFL_3484 | sigma factor regulatory protein, putative | 1.14 | NS | 0.38 | — | — | — | — | — |
| PFL_3490 | enantio-pyochelin biosynthetic protein PchC | 1.21 | NS | NS | PA4229 | 6.95 | 4.60 | — | — |
| PFL_3491 | saccharopine dehydrogenase PchK | 1.30 | NS | NS | — | — | — | — | — |
| PFL_3492 | enantio-pyochelin synthetase PchF | 1.11 | NS | NS | PA4225 | 5.21 | 4.79 | — | — |
| PFL_3495 | ABC transporter, permease/ATP-binding protein, putative, PchH | 1.60 | NS | NS | — | — | — | PSPTO2604 | 7.14 |
| PFL_3496 | salicyl-AMP ligase PchD | 1.11* | 0.45* | NS | PA4228 | 6.95 | 4.68 | PSPTO2597 | 8.26 |
| PFL_3497 | regulatory protein PchR, | 3.41 | 1.53 | 1.38 | PA4227 | 5.21 | NS | — | — |
| PFL_3498 | TonB-dependent outermembrane enantio-pyochelin receptor FetA | 2.01 | NS | NS | — | — | — | — | — |
| PFL_3499 | PepSY-associated membrane protein | 1.70 | NS | NS | — | — | — | — | — |
| PFL_3500 | iron-chelate uptake ABC transporter, FeCT family, periplasmic iron-chelate-binding protein, putative | 1.87 | NS | NS | — | — | — | — | — |
| PFL_3501 | iron-chelate uptake ABC transporter, FeCT family, permease protein | 1.23 | NS | NS | — | — | — | — | — |
| PFL_3502 | iron-chelate uptake ABC transporter, FeCT family, ATP-binding protein | 1.23 | NS | NS | — | — | — | — | — |
| PFL_3503 | transporter, major facilitator family | 1.06 | NS | NS | — | — | — | — | — |
| PFL_3583 | endoribonuclease L-PSP family protein | 1.10 | NS | NS | — | — | — | PSPTO2779 | NS |
| PFL_3594 | transcriptional regulator, LysR family | 1.09 | NS | NS | — | — | — | — | — |
| PFL_3618 | molybdenum-pterin-binding protein | 1.12 | NS | NS | — | — | — | — | — |
| PFL_3619 | monooxygenase, NtaA/SnaA/SoxA family | 1.56 | NS | 0.38 | PA4155 | NS | NS | — | — |
| PFL_3620 | TonB-dependent receptor | 1.39 | NS | NS | PA4156 | NS | 4.85 | — | — |
| PFL_3623^ | ferric enterobactin ABC transporter, periplasmic ferric enterobactin-binding protein FepB | 2.74 | NS | 0.77 | PA4159 | NS | NS | — | — |
| PFL_3624 | ferric enterobactin ABC transporter, permease protein FepD | 1.53 | NS | 0.26 | PA4160 | NS | NS | PSPTO0762 | NS |
| PFL_3636 | response regulator/EAL domain protein | 1.06 | NS | NS | — | — | — | — | — |
| PFL_3804 | transcriptional regulator, LysR family | 2.01* | 0.81* | NS | PA1141 | NS | NS | — | — |
| PFL_3806^ | ribosomal protein L36 RpmJ | 3.20 | 2.67 | 0.14 | PA3600 | NS | NS | PSPTO0647 | NS |
| PFL_3864 | hypothetical protein | 1.20 | NS | NS | — | — | — | — | — |
| PFL_3941 | hypothetical protein | 2.24 | NS | NS | — | — | — | — | — |
| PFL_3995 | hypothetical protein | 1.89 | NS | NS | — | — | — | PSPTO2692 | NS |
| PFL_4026 | oligopeptide/dipeptide ABC transporter, permease protein | 1.18 | NS | NS | — | — | — | PSPTO3250 | NS |
| PFL_4041^ | RNA polymerase sigma-70 factor, ECF subfamily | 1.61 | 0.73 | 1.43 | — | — | — | — | — |
| PFL_4043 | DNA-binding protein | 1.02 | NS | NS | — | — | — | — | — |
| PFL_4079 | L-ornithine 5-monooxygenase PvdA | 1.81 | NS | 2.85 | PA2386 | 7.75 | 2.70 | — | — |
| PFL_4080^ | RNA polymerase sigma-70 factor, ECF subfamily, FpvI | 2.84* | 1.02* | 2.51 | PA2387 | 2.32 | 2.63 | — | — |
| PFL_4082 | efflux ABC transporter, ATP-binding/permease protein | 1.09 | NS | NS | PA2390 | 3.81 | NS | PSPTO2159 | NS |
| PFL_4083 | efflux transporter, outer membrane factor lipoprotein, NodT family | 1.09 | NS | NS | PA2391 | 3.81 | NS | PSPTO2158 | NS |
| PFL_4086 | chromophore maturation protein PvdP | 1.15 | NS | 2.17 | PA2392 | 3.81 | NS | PSPTO5624 | NS |
| PFL_4093 | non-ribosomal peptide synthetase PvdD | 1.74 | NS | NS | — | — | — | PSPTO2150 | 7.08 |
| PFL_4095 | non-ribosomal peptide synthetase PvdI | 1.19 | NS | NS | — | — | — | — | — |
| PFL_4096 | siderophore-interacting protein | 1.17 | 1.17 | 2.38 | PA2033 | 6.57 | 4.10 | — | — |
| PFL_4159 | antibiotic biosynthesis monooxygenase | 1.02 | NS | NS | — | — | — | — | — |
| PFL_4169 | PepSY-associated TM helix domain protein | 2.39 | 1.48 | 1.00 | PA2403 | 3.91 | 2.23 | PSPTO2145 | 6.44 |
| PFL_4171 | hypothetical protein | 1.44 | NS | NS | PA2405 | 3.91 | 2.41 | PSPTO2143 | 4.33 |
| PFL_4178 | MbtH-like protein | 3.99 | 4.73 | 2.77 | — | — | — | PSPTO2137 | 9.02 |
| PFL_4189^ | non-ribosomal peptide synthetase PvdL | 2.59 | NS | NS | PA2424 | 5.09 | 1.49 | PSPTO2135 | 7.40 |
| PFL_4190^ | polymerase sigma-70 factor, ECF subfamily, PvdS | 5.07 | 3.94 | 4.09 | PA2426 | 6.87 | 3.69 | PSPTO2133 | 8.63 |
| PFL_4273 | hypothetical protein | 1.01 | NS | NS | — | — | — | — | — |
| PFL_4339 | hypothetical protein | NS | 1.71 | NS | — | — | — | — | — |
| PFL_4449 | sensor histidine kinase | 1.27 | 0.49 | 0.49 | PA0930 | 2.58 | NS | — | — |
| PFL_4450 | DNA-binding response regulator | 3.22 | 1.09 | NS | PA0929 | 2.58 | NS | — | — |
| PFL_4464 | hypothetical protein | 1.69 | NS | NS | PA1190 | NS | NS | — | — |
| PFL_4467 | aerobic C4-dicarboxylate transport protein DctA | 1.24 | NS | NS | PA1183 | NS | NS | — | — |
| PFL_4476 | ribonucleoside-diphosphate reductase, alpha subunit | 1.01 | NS | NS | PA1156 | NS | NS | PSPTO1671 | NS |
| PFL_4522 | acetate--CoA ligase AcsA_1 | 1.93 | NS | 0.38 | PA0887 | NS | NS | PSPTO1825 | NS |
| PFL_4625^ | RNA polymerase sigma-70 factor, ECF subfamily | 1.70 | 0.85 | 1.07 | — | — | — | PSPTO1286 | NS |
| PFL_4628 | heme oxygenase HemO | 2.28 | NS | 2.87 | PA0672 | 7.11 | NS | PSPTO1283 | 4.84 |
| PFL_4817 | hypothetical protein | 1.06 | NS | NS | — | — | — | — | — |
| PFL_4818 | transcriptional regulator, AraC family | 1.71 | NS | 1.81 | PA0248 | NS | NS | — | — |
| PFL_4828^ | insulin-cleaving metalloproteinase outer membrane protein IcmP | 2.12 | NS | 1.85 | PA4370 | 4.25 | 4.15 | PSPTO4366 | 2.89 |
| PFL_4839 | chaperonin GroS | NS | 1.76 | 1.20 | PA4386 | NS | NS | PSPTO4377 | NS |
| PFL_4840 | phage T7 F exclusion suppressor FxsA | 0.69 | 1.66 | 1.38 | PA4387 | NS | NS | PSPTO4379 | -0.64 |
| PFL_4841 | pyridoxamine 5'-phosphate oxidase family protein | NS | 1.22 | 1.26 | PA4388 | NS | NS | — | — |
| PFL_4858^ | bacterioferritin-associated ferredoxin, putative | NS | 4.83 | 1.96 | PA3530 | 7.67 | 4.15 | PSPTO4159 | 8.67 |
| PFL_4886 | hypothetical protein | NS | 1.51 | 0.93 | — | — | — | — | — |
| PFL_4895 | response regulator/HD domain protein | 1.48 | 1.10 | NS | — | — | — | — | — |
| PFL_4907 | pseudo | 1.05 | NS | NS | — | — | — | — | — |
| PFL_4944 | exodeoxyribonuclease VII, large subunit XseA | 1.55 | NS | 0.26 | PA3777 | NS | NS | PSPTO1446 | NS |
| PFL_4966 | iron-sulfur cluster assembly transcription factor IscR | NS | 1.29 | NS | PA3815 | NS | NS | PSPTO1422 | NS |
| PFL_4993 | outer membrane porin, OprD family | 1.35 | NS | NS | PA0189 | NS | NS | — | — |
| PFL_4995 | hypothetical protein | 1.09 | 0.89 | NS | — | — | — | — | — |
| PFL_5037 | cytochrome o ubiquinol oxidase, subunit II, CyoA_2 | NS | 1.55 | NS | PA1317 | 2.00 | NS | PSPTO1325 | NS |
| PFL_5043 | acetyltransferase, GNAT family | 1.57 | NS | NS | — | — | — | PSPTO1322 | NS |
| PFL_5091 | metallo-beta-lactamase domain protein | 1.39 | 1.12 | NS | PA0832 | NS | NS | — | — |
| PFL_5131 | hypothetical protein | 1.83 | NS | NS | — | — | — | PSPTO1139 | NS |
| PFL_5145 | hypothetical protein | 1.05 | NS | NS | PA4658 | NS | NS | PSPTO1123 | NS |
| PFL_5266^ | hemin ABC transporter, periplasmic hemin-binding protein PhuT | 1.01 | 0.37 | 1.38 | PA4708 | 3.58 | NS | — | — |
| PFL_5332^ | riboflavin biosynthesis protein RibF | 4.72 | 4.32 | 2.00 | PA4570 | 8.65 | NS | PSPTO4580 | 6.28 |
| PFL_5337 | hypothetical protein | 2.04 | NS | NS | — | — | — | PSPTO4584 | NS |
| PFL_5377 | heme acquisition protein HasAp | 3.70 | NS | NS | PA3407 | 6.13 | NS | — | — |
| PFL_5379 | sigma factor regulatory protein HasS | 2.74 | NS | 0.85 | PA3409 | 5.32 | NS | — | — |
| PFL_5380^ | RNA polymerase sigma-70 factor, ECF subfamily, HasI | 1.72 | NS | 1.32 | PA3410 | 5.32 | 3.72 | — | — |
| PFL_5438 | hypothetical protein | 2.66* | 0.91* | NS | — | — | — | PSPTO4810 | NS |
| PFL_5693 | biotin synthetase BioB | 1.69 | NS | NS | PA0500 | 2.32 | NS | PSPTO0494 | NS |
| PFL_5703 | exonuclease | 1.03 | NS | NS | — | — | — | PSPTO0470 | NS |
| PFL_5704^ | RNA polymerase sigma-70 factor, ECF subfamily | 3.10 | 0.98 | 1.68 | PA0472 | 5.52 | 3.57 | PSPTO0444 | NS |
| PFL_5705 | sigma factor regulatory protein, FecR/PupR family | 3.36 | NS | NS | PA0471 | 5.52 | 4.28 | PSPTO0445 | NS |
| PFL_5755 | hypothetical protein | 1.00 | NS | NS | PA5385 | NS | NS | — | — |
| PFL_5787 | ArsR family transcriptional regulator | 1.10 | NS | NS | PA0547 | NS | NS | PSPTO0384 | NS |
| PFL_5953 | transcriptional repressor PhlF | 2.42 | NS | NS | — | — | — | — | — |
| PFL_5954 | 2,4-diacetylphloroglucinol biosynthesis protein PhlA | 2.44 | NS | NS | — | — | — | — | — |
| PFL_5964^ | ferric iron ABC transporter, FeT family, periplasmic ferric iron-binding protein, putative | 1.78 | NS | NS | PA5217 | 4.17 | 3.32 | PSPTO0314 | 3.21 |
| PFL_6046 | aldehyde dehydrogenase family protein | 1.05 | NS | NS | PA5312 | 2.00 | 1.14 | PSPTO0092 | NS |
| PFL_6066 | ActC family protein | 1.33 | NS | 0.93 | PA5343 | NS | NS | PSPTO0070 | NS |
| PFL_6067 | TonB system transport protein ExbB1 | 3.40 | NS | 2.20 | — | — | — | PSPTO0069 | 8.88 |
| PFL_6068 | TonB system transport protein ExbD1 | 2.85 | NS | 2.07 | — | — | — | PSPTO0068 | 6.47 |
| PFL_6069 | periplasmic energy transduction protein TonB1 | 3.55 | 1.52 | 2.68 | — | — | — | PSPTO0067 | 5.24 |

^ Pf-5 genes that contain Fur binding motifs upstream

* Values are statistically different as determined using MultiExperiment Viewer (MeV) 4.1 with P-value < 0.01

NS means not significant when analyzed with SAM in this study as well as the study comparing a *gacA* mutant to the parental strain Pf-5 [35]. Similarly, for the studies on *P. aeruginosa* [27], [28] and on *P. syringae* DC3000 [29], NS means not significant when analyzed according to their respective study criteria.

(B) Genes whose transcripts were down-regulated under iron limitation conditions and comparison with Pf-5 *gacA* mutant study [35], *P. aeruginosa* PAO1 iron limitation studies (performed by Ochsner

et al. [27] and Palma et al. [28]), and *P. syringae* DC3000 iron limitation study (performed by Bronstein et al. [29]).

| Gene ID | Annotated function | – FeCl_2_ | – FeCl_3_ | *gacA* mutant [35] | PAO1 ortho-logues | Ochsner  et al. [27] | Palma  et al. [28] | DC3000 orthologues | Bronstein  et al. [29] |
| --- | --- | --- | --- | --- | --- | --- | --- | --- | --- |
| PFL_0080 | homoserine kinase ThrB | NS | -1.46 | NS | PA5495 | — | NS | PSPTO0346 | NS |
| PFL_0084 | cytochrome c4 | -1.18* | -0.46* | -1.54 | PA5490 | — | NS | — | — |
| PFL_0097 | lipoprotein | -1.26 | -0.87 | NS | PA3214 | — | NS | — | — |
| PFL_0112 | succinate CoA transferase | NS | -1.26 | NS | PA5445 | — | NS | PSPTO5465 | NS |
| PFL_0137 | taurine dioxygenase, TauD/TfdA family | NS | -1.23 | NS | PA2310 | — | NS | PSPTO5198 | NS |
| PFL_0141 | transcriptional regulator, LysR family | NS | -1.46 | NS | PA2834 | — | NS | — | — |
| PFL_0157 | auxiliary transport protein, membrane fusion protein family | -1.42 | NS | NS | — | — | — | PSPTO3620 | NS |
| PFL_0192 | sulfate ABC transporter, periplasmic sulfate-binding protein Sbp | -1.68 | -1.20 | NS | PA0283 | — | NS | PSPTO0308 | NS |
| PFL_0194 | sulfate ABC transporter, permease protein CysW | -1.54 | NS | -0.26 | PA0281 | — | NS | PSPTO0310 | NS |
| PFL_0225 | TonB2 protein | NS | -2.83 | NS | PA0197 | — | NS | PSPTO2481 | NS |
| PFL_0226 | TonB system transport protein ExbB2 | -0.99 | -2.35 | NS | PA0198 | — | NS | PSPTO2482 | NS |
| PFL_0228 | hypothetical protein | -0.69 | -1.42 | NS | PA0201 | — | NS | PSPTO5218 | NS |
| PFL_0229 | sigma-54-binding protein | -1.07 | -1.43 | -0.26 | PA3932 | — | NS | PSPTO5217 | NS |
| PFL_0233 | amino acid ABC transporter, ATP-binding protein | -1.10 | -0.83 | NS | — | — | — | PSPTO3083 | NS |
| PFL_0234 | amino acid ABC transporter, periplasmic amino acid-binding protein, putative | -1.21 | NS | -0.77 | — | — | — | PSPTO3082 | NS |
| PFL_0248 | polar amino acid ABC transporter, periplasmic amino acid-binding protein | -0.43* | -1.21* | NS | — | — | — | PSPTO5180 | NS |
| PFL_0263 | fatty acid desaturase family protein | -1.04 | NS | -3.17 | — | — | — | — | — |
| PFL_0267 | hypothetical protein | NS | -1.22 | -2.10 | — | — | — | — | — |
| PFL_0268 | dihydrolipoamide dehydrogenase LpdA_2 | -0.99 | -1.62 | -2.26 | — | — | — | — | — |
| PFL_0271 | taurine ABC transporter, ATP-binding protein TauB | NS | -1.44 | NS | PA3937 | — | NS | PSPTO5320 | NS |
| PFL_0272 | taurine ABC transporter, periplasmic binding protein TauA | NS | -1.43 | NS | PA3938 | — | NS | PSPTO5319 | NS |
| PFL_0398 | formiminoglutamate deiminase HutF | -1.21 | NS | NS | PA5106 | — | NS | PSPTO5171 | NS |
| PFL_0448 | type IV pilus biogenesis protein PilP_1 | -1.17 | NS | NS | PA5041 | — | NS | PSPTO5129 | NS |
| PFL_0592 | azurin Azu | NS | -1.24 | -1.49 | PA4922 | — | NS | PSPTO4923 | 2.38 |
| PFL_0649 | hypothetical protein | -1.40 | NS | NS | — | — | — | PSPTO4882 | NS |
| PFL_0650 | hypothetical protein | -1.14 | NS | NS | PA3785 | — | NS | PSPTO4881 | NS |
| PFL_0673 | 3-dehydroquinate dehydratase, type II AroQ_2 | -1.33 | NS | NS | PA4846 | — | NS | PSPTO4859 | NS |
| PFL_0762 | kynureninase KynU | -1.34 | NS | NS | PA2080 | — | -1.5 | — | — |
| PFL_0936 | sulfate adenylate transferase, large subunit/adenylylsulfate kinase, putative CysN | -1.21* | -0.42* | -0.49 | PA4442 | — | NS | PSPTO4432 | NS |
| PFL_0957 | LrgA family protein | -1.62 | -0.76 | NS | PA3432 | — | NS | — | — |
| PFL_0958 | LrgB family membrane protein | -2.00 | NS | NS | PA3431 | — | NS | — | — |
| PFL_0959 | class II aldolase and adducin N-terminal domain protein | -1.07 | NS | NS | PA3430 | — | NS | — | — |
| PFL_1000 | hypothetical protein | -1.30 | NS | NS | PA3897 | — | NS | PSPTO1214 | NS |
| PFL_1229 | putidacin L1 LlpA1 | -1.11* | -0.46* | NS | — | — | — | — | — |
| PFL_1284^ | hypothetical protein | NS | -1.03 | NS | — | — | — | — | — |
| PFL_1394 | lipoprotein | -1.08 | -0.77 | NS | — | — | — | — | — |
| PFL_1395 | dihydrodipicolinate synthase, putative | -1.01 | NS | NS | PA0223 | — | -2.8 | — | — |
| PFL_1399 | alcohol dehydrogenase II AdhB | -1.10 | -1.46 | -2.10 | PA1991 | — | NS | PSPTO4285 | NS |
| PFL_1520 | Aspartyl/Asparaginyl beta-hydroxylase family | -0.77 | -1.04 | NS | PA0936 | — | NS | PSPTO3901 | NS |
| PFL_1613 | flagellar basal-body rod protein FlgF | -1.30 | NS | NS | PA1081 | — | NS | PSPTO1939 | NS |
| PFL_1614 | flagellar basal-body rod protein FlgG | -1.34 | NS | NS | PA1082 | — | NS | PSPTO1940 | NS |
| PFL_1616 | flagellar P-ring protein FlgI | -1.13 | NS | NS | PA1084 | — | NS | PSPTO1942 | NS |
| PFL_1636 | sigma-54 dependent DNA-binding response regulator FleR | -1.41 | -1.16 | NS | PA1099 | — | NS | PSPTO1956 | NS |
| PFL_1637 | flagellar hook-basal body complex protein FliE | -1.66 | NS | NS | PA1100 | — | NS | PSPTO1957 | NS |
| PFL_1638 | flagellar M-ring protein FliF | -1.74* | -0.87* | NS | PA1101 | — | NS | PSPTO1958 | NS |
| PFL_1639 | flagellar motor switch protein FliG | -1.75* | -0.70* | -0.26 | PA1102 | — | NS | PSPTO1959 | NS |
| PFL_1640 | Flagellar assembly protein FliH | -1.18 | NS | NS | PA1103 | — | NS | PSPTO1960 | NS |
| PFL_1641 | flagellum-specific ATP synthase FliI | -1.12 | NS | NS | PA1104 | — | NS | PSPTO1961 | NS |
| PFL_1646 | flagellar hook-length control protein FliK | -1.23 | NS | NS | PA1441 | — | NS | PSPTO1966 | NS |
| PFL_1647 | flagellar protein FliL | -1.38 | NS | NS | PA1442 | — | NS | PSPTO1968 | NS |
| PFL_1648 | flagellar motor switch protein FliM | -1.47 | NS | NS | PA1443 | — | NS | PSPTO1969 | NS |
| PFL_1649 | flagellar motor switch protein FliN | -1.96 | NS | NS | PA1444 | — | NS | PSPTO1970 | NS |
| PFL_1652 | flagellar biosynthetic protein FliQ | -1.04 | NS | NS | PA1447 | — | NS | PSPTO1973 | NS |
| PFL_1656 | hypothetical protein | -1.87 | NS | NS | — | — | — | — | — |
| PFL_1657 | 3-oxoacyl-ACP synthase | -1.32 | -0.68 | NS | — | — | — | — | — |
| PFL_1664 | flagellar biosynthesis protein FlhA | -1.48 | NS | NS | PA1452 | — | NS | PSPTO1976 | NS |
| PFL_1666 | flagellar synthesis regulator FleN | NS | -1.55 | -1.72 | PA1454 | — | NS | PSPTO1978 | NS |
| PFL_1667 | motility sigma factor FliA | NS | -1.39 | -1.07 | PA1455 | — | NS | PSPTO1979 | NS |
| PFL_1683 | heme exporter protein CcmD | -1.58 | -1.18 | -1.20 | — | — | — | PSPTO3632 | NS |
| PFL_1684 | cytochrome c-type biogenesis protein CcmE | -1.24 | NS | -0.77 | PA1479 | — | NS | PSPTO3631 | NS |
| PFL_1685 | cytochrome c-type biogenesis protein CcmF | -1.04 | NS | -0.58 | PA1480 | — | NS | PSPTO3630 | NS |
| PFL_1687 | cytochrome c-type biogenesis protein CycL | -1.10 | NS | -0.49 | PA1482 | — | NS | PSPTO3628 | NS |
| PFL_1795 | malonyl CoA-acyl carrier protein transacylase FabD | -1.01* | 0.62* | NS | PA2968 | — | NS | PSPTO3833 | -0.46 |
| PFL_1835 | acetyl-CoA C-acetyltransferase | -1.05 | NS | NS | PA1736 | — | NS | — | — |
| PFL_1836 | 3-hydroxyacyl-CoA dehydrogenase, putative | -1.14 | NS | NS | PA1737 | — | NS | — | — |
| PFL_1912 | oxygen-independent coproporphyrinogen III oxidase HemN | -1.23 | -1.52 | -2.04 | PA1546 | — | -1.0 | PSPTO1993 | NS |
| PFL_1915 | copper-translocating P-type ATPase | -1.31 | NS | NS | PA1549 | — | NS | PSPTO1996 | NS |
| PFL_1916 | conserved hypothetical protein | -1.37 | NS | -1.43 | PA1550 | — | NS | PSPTO1997 | NS |
| PFL_1917 | cytochrome c oxidase accessory protein CcoG | -1.58 | -0.72 | -1.89 | PA1551 | — | NS | PSPTO1998 | NS |
| PFL_1922 | cytochrome c oxidase, cbb3-type, subunit III, CcoP_2 | NS | -1.29 | -2.41 | — | — | — | — | — |
| PFL_1923 | cytochrome c oxidase, cbb3-type, CcoQ subunit CcoQ_2 | -1.00 | -1.36 | -2.83 | — | — | — | — | — |
| PFL_1924 | cytochrome c oxidase, cbb3-type, subunit II, CcoO_2 | -1.57 | -1.47 | -2.66 | — | — | — | — | — |
| PFL_1925 | cytochrome c oxidase, cbb3-type, subunit I, CcoN_2 | NS | -1.57 | -2.66 | PA1557 | — | NS | — | — |
| PFL_1929 | aconitate hydratase 1 AcnA | -1.44 | NS | NS | PA1562 | — | NS | PSPTO2016 | NS |
| PFL_1934 | hypothetical protein | NS | -1.05 | NS | — | — | — | PSPTO2020 | NS |
| PFL_1938 | universal stress protein family protein | NS | -1.31 | NS | PA3017 | — | NS | PSPTO3520 | NS |
| PFL_1968 | hypothetical protein | NS | -1.00 | -0.85 | — | — | — | PSPTO2113 | NS |
| PFL_2099 | glutamin-(asparagin-)ase ansB | -1.38 | NS | -1.43 | PA1337 | — | NS | — | — |
| PFL_2121 | hypothetical protein | NS | -1.74 | NS | — | — | — | — | — |
| PFL_2134 | transporter, major facilitator family | -1.82 | NS | NS | PA5219 | — | NS | — | — |
| PFL_2135 | hypothetical protein | -1.09 | -0.96 | NS | PA5220 | — | NS | — | — |
| PFL_2349 | hypothetical protein | -2.22 | NS | NS | — | — | — | — | — |
| PFL_2350 | pseudo | -2.33 | NS | NS | — | — | — | — | — |
| PFL_2351 | hypothetical protein | -1.76 | NS | NS | — | — | — | — | — |
| PFL_2522 | hypothetical protein | -2.80 | NS | NS | — | — | — | — | — |
| PFL_2552 | QAT family ABC transporter substrate-binding protein | NS | -1.26 | NS | PA2594 | — | NS | PSPTO2423 | NS |
| PFL_2577 | hydrogen cyanide synthase HcnA | NS | -1.49 | -3.46 | PA2193 | — | NS | — | — |
| PFL_2578 | hydrogen cyanide synthase HcnB | -1.26 | -1.49 | -3.15 | PA2194 | — | NS | — | — |
| PFL_2579 | hydrogen cyanide synthase HcnC | NS | -1.36 | -2.38 | PA2195 | — | NS | — | — |
| PFL_2597 | methyl-accepting chemotaxis protein | NS | -1.11 | NS | PA4290 | — | NS | PSPTO3480 | NS |
| PFL_2681 | hypothetical protein | NS | -1.63 | NS | — | — | — | — | — |
| PFL_2682 | hypothetical protein | -0.95 | -1.11 | NS | PA3765 | — | NS | — | — |
| PFL_2795 | membrane fusion protein, putative PltI | -1.75 | NS | NS | — | — | — | — | — |
| PFL_2833 | hypothetical protein | -1.20 | NS | NS | — | — | — | — | — |
| PFL_2834 | cytochrome c oxidase, cbb3-type, subunit I, CcoN_3 | -1.57 | -0.74 | -2.49 | PA4133 | — | NS | — | — |
| PFL_2835 | sulfite reductase (NADPH) hemoprotein, beta-component CysI_1 | -1.45 | NS | -2.63 | PA4130 | — | NS | — | — |
| PFL_2836 | hypothetical protein | -1.22 | NS | -2.23 | PA4129 | — | NS | — | — |
| PFL_2869 | cytochrome c oxidase accessory protein CcoG | -1.04 | NS | -2.70 | PA4131 | — | NS | — | — |
| PFL_2909 | nitrilase family protein | -1.55 | NS | NS | — | — | — | — | — |
| PFL_2917 | oxidoreductase membrane protein, FAD-binding | -1.93 | -2.31 | NS | — | — | — | — | — |
| PFL_3190 | oxidoreductase, 2OG-Fe(II) oxygenase family | NS | -1.20 | NS | — | — | — | — | — |
| PFL_3191 | TOBE domain protein | NS | -1.55 | -0.26 | — | — | — | — | — |
| PFL_3348 | hypothetical protein | NS | -1.34 | 2.04 | — | — | — | — | — |
| PFL_3555 | amidohydrolase family protein | NS | -1.26 | NS | — | — | — | — | — |
| PFL_3819 | cyclic diguanylate phosphodiesterase (EAL) domain protein | -1.72 | NS | NS | PA2133 | — | NS | — | — |
| PFL_3903 | NADH-quinone oxidoreductase, H subunit NuoH | -1.01 | NS | NS | PA2643 | — | NS | PSPTO3371 | -2.87 |
| PFL_3904 | NADH-quinone oxidoreductase, I subunit NuoI | -1.03 | NS | NS | PA2644 | — | NS | PSPTO3372 | NS |
| PFL_3906 | NADH-quinone oxidoreductase, K subunit nuoK | -1.09 | NS | NS | PA2646 | — | NS | PSPTO3374 | NS |
| PFL_3907 | NADH-quinone oxidoreductase, L subunit nuoL | -1.05 | NS | NS | PA2647 | — | NS | PSPTO3375 | NS |
| PFL_3971 | Fatty acid desaturase domain protein | -1.16 | -0.61 | NS | — | — | — | — | — |
| PFL_4067 | universal stress protein family protein | NS | -1.14 | -1.77 | — | — | — | PSPTO1667 | NS |
| PFL_4068 | alcohol dehydrogenase, zinc-containing Adh | NS | -1.78 | NS | PA2119 | — | NS | — | — |
| PFL_4303 | oxidoreductase, short chain dehydrogenase/reductase family | -1.25 | -1.78 | -1.81 | PA4832 | — | NS | — | — |
| PFL_4305 | nucleotide sugar epimerase/dehydratase WbpM | -0.68 | -1.08 | NS | PA3141 | — | NS | PSPTO1756 | NS |
| PFL_4410 | C4-dicarboxylate transporter/malic acid transport protein | -1.58 | NS | NS | — | — | — | PSPTO4613 | NS |
| PFL_4480 | flagellar basal-body rod protein FlgB | -1.08 | NS | NS | PA1077 | — | NS | PSPTO1933 | NS |
| PFL_4542 | peptidase propeptide and YPEB domain protein | NS | -1.69 | NS | — | — | — | PSPTO1609 | -0.76 |
| PFL_4546 | low-affinity inorganic phosphate transporter PitA_2 | NS | -1.83 | NS | PA0450 | — | NS | PSPTO3702 | NS |
| PFL_4608 | 2-dehydro-3-deoxyphosphogluconate aldolase/4-hydroxy-2-oxoglutarate aldolase Eda | -1.41 | NS | -0.68 | PA3181 | — | NS | PSPTO1302 | NS |
| PFL_4609 | 6-phosphogluconolactonase Pgl | -1.68 | NS | -0.77 | PA3182 | — | NS | PSPTO1301 | NS |
| PFL_4610 | glucose-6-phosphate 1-dehydrogenase Zwf_2 | -1.50 | -1.21 | NS | PA3183 | — | NS | PSPTO1300 | NS |
| PFL_4622 | phosphogluconate dehydratase Edd | -1.06 | NS | NS | PA3194 | — | NS | PSPTO1288 | NS |
| PFL_4624 | methylglyoxal synthase MgsA | -1.31 | -0.90 | NS | — | — | — | — | — |
| PFL_4632 | DNA-3-methyladenine glycosylase II AlkA | -1.54 | NS | NS | PA1686 | — | NS | — | — |
| PFL_4633 | arginine/ornithine antiporter ArcD2 | -1.03 | -1.34 | NS | — | — | — | — | — |
| PFL_4634 | arginine/ornithine antiporter ArcD | NS | -2.16 | -1.93 | PA5170 | — | NS | — | — |
| PFL_4635 | arginine deiminase ArcA | -1.50 | -1.75 | -2.04 | PA5171 | — | NS | — | — |
| PFL_4636 | ornithine carbamoyltransferase ArgF_1 | -1.54 | -1.06 | -1.49 | PA5172 | — | NS | — | — |
| PFL_4637 | carbamate kinase ArcC | -1.83 | -1.06 | NS | PA5173 | — | NS | — | — |
| PFL_4638 | hypothetical protein | -1.00 | NS | NS | — | — | — | PSPTO1281 | NS |
| PFL_4684 | hypothetical protein | -1.63 | NS | -0.68 | — | — | — | — | — |
| PFL_4686 | hypothetical protein | -1.20 | NS | NS | — | — | — | — | — |
| PFL_4826 | superoxide dismutase (Fe) SodB | -1.53 | -1.13 | -1.38 | PA4366 | — | NS | PSPTO4363 | -2.36 |
| PFL_4845 | hypothetical protein | NS | -1.33 | NS | — | — | — | — | — |
| PFL_4904 | hypothetical protein | -1.36 | NS | NS | — | — | — | — | — |
| PFL_4912 | TonB-dependent outer membrane receptor | NS | -1.21 | NS | PA0781 | — | NS | — | — |
| PFL_4986 | polyphosphate kinase 2 Ppk2 | NS | -1.50 | -2.54 | PA0141 | — | NS | — | — |
| PFL_4987 | hypothetical protein | -1.33 | NS | NS | — | — | — | — | — |
| PFL_4990 | glutathione S-transferase, putative | -1.84 | NS | -2.41 | PA4401 | — | NS | PSPTO4398 | NS |
| PFL_5004 | cytochrome c family protein | -1.18 | -1.17 | -2.17 | PA4571 | — | NS | — | — |
| PFL_5056 | cell division protein FtsZ | -1.25 | NS | NS | PA4407 | — | NS | PSPTO4403 | NS |
| PFL_5057 | cell division protein FtsA | -1.07 | NS | NS | PA4408 | — | NS | PSPTO4404 | NS |
| PFL_5060 | UDP-N-acetylmuramate--alanine ligase MurC | -1.15 | NS | NS | PA4411 | — | NS | PSPTO4407 | NS |
| PFL_5061 | undecaprenyldiphospho-muramoylpentapeptide beta-N-acetylglucosaminyltransferase MurG | -1.05 | NS | NS | PA4412 | — | NS | PSPTO4408 | NS |
| PFL_5062 | cell division protein FtsW | -0.91 | -1.06 | NS | PA4413 | — | NS | PSPTO4409 | NS |
| PFL_5069 | S-adenosyl-methyltransferase MraW | NS | -1.01 | NS | PA4420 | — | NS | PSPTO4416 | NS |
| PFL_5070 | cell division protein MraZ | NS | -1.05 | NS | PA4421 | — | NS | PSPTO4417 | NS |
| PFL_5078 | ubiquinol-cytochrome c reductase, cytochrome c1, putative | -1.20 | NS | -0.77 | PA4429 | — | NS | — | — |
| PFL_5080 | ubiquinol-cytochrome c reductase, iron-sulfur subunit, PetA | -1.41 | NS | -1.43 | PA4431 | — | NS | — | — |
| PFL_5183 | cytochrome c551 peroxidase CcpA_2 | NS | -2.63 | -1.58 | — | — | — | — | — |
| PFL_5292 | hypothetical protein | -0.68* | -2.17* | -1.07 | — | — | — | — | — |
| PFL_5404 | hypothetical protein | NS | -1.07 | NS | — | — | — | — | — |
| PFL_5422 | hypothetical protein | NS | -1.08 | NS | — | — | — | — | — |
| PFL_5489 | hypothetical protein | -1.06 | NS | NS | — | — | — | — | — |
| PFL_5501 | copper-containing nitrite reductase NirK | -1.59 | NS | NS | — | — | — | — | — |
| PFL_5532 | outer membrane protein OprG | NS | -1.38 | -3.04 | PA4067 | — | NS | PSPTO0679 | NS |
| PFL_5555 | bacterioferritin A Bfr_2 | -2.48* | -0.72* | -1.54 | PA4235 | — | NS | PSPTO0653 | -4.39 |
| PFL_5556 | catalase KatA | -1.37 | -0.70 | -0.38 | PA4236 | — | NS | — | — |
| PFL_5612 | hypothetical protein | -1.10 | NS | -0.68 | PA0661 | — | NS | PSPTO0603 | -1.33 |
| PFL_5655 | SpoVR family protein | NS | -1.04 | -1.07 | PA0586 | — | NS | PSPTO0545 | NS |
| PFL_5687 | acyl-CoA dehydrogenase family protein | -1.68 | NS | NS | PA0506 | — | NS | PSPTO0500 | NS |
| PFL_5775 | hypothetical protein | NS | -1.08 | NS | — | — | — | PSPTO5010 | NS |
| PFL_5798 | adenosylhomocysteinase AhcY | -1.01 | NS | NS | PA0432 | — | NS | PSPTO5068 | NS |
| PFL_5844 | hypothetical protein | NS | -1.30 | NS | PA0388 | — | NS | — | — |
| PFL_5845 | non-canonical purine NTP pyrophosphatase RdgB | NS | -1.29 | NS | PA0387 | — | NS | PSPTO5051 | NS |
| PFL_5933 | molybdenum-pterin-binding protein SsuF | -1.76 | NS | NS | — | — | — | PSPTO5313 | NS |
| PFL_5935 | aliphatic sulfonates ABC transporter, permease protein SsuC | -0.99 | -1.13 | NS | PA3443 | — | NS | PSPTO5315 | NS |
| PFL_5937 | aliphatic sulfonates ABC transporter, periplasmic sulfonate-binding protein SsuA | -0.68 | -1.35 | -0.26 | PA3445 | — | NS | PSPTO5316 | NS |
| PFL_5938 | NADH-dependent FMN reductase SsuE | NS | -1.23 | NS | PA3446 | — | NS | PSPTO3451 | NS |
| PFL_5939 | thiol-specific antioxidant protein LsfA | -1.28 | -1.17 | -0.77 | PA3450 | — | NS | PSPTO5317 | NS |
| PFL_5965 | 2-octaprenyl-3-methyl-6-methoxy-1,4-benzoquinol hydroxylase | -1.54 | -1.10 | NS | PA5221 | — | NS | PSPTO5221 | NS |
| PFL_5966 | hypothetical protein | -1.07 | -1.05 | NS | PA5222 | — | NS | — | — |
| PFL_5967 | 2-polyprenyl-6-methoxyphenol 4-hydroxylase UbiH | -0.83 | -1.23 | NS | PA5223 | — | NS | PSPTO5222 | NS |
| PFL_5968 | Xaa-Pro aminopeptidase PepP | -0.82* | -1.62* | -0.68 | PA5224 | — | NS | PSPTO5223 | NS |
| PFL_5973 | auxiliary transport protein, membrane fusion protein family | NS | -1.17 | NS | PA5232 | — | NS | — | — |
| PFL_5974 | ABC transporter, ATP binding/permease protein, putative | -1.38* | -0.72* | NS | PA5231 | — | NS | — | — |
| PFL_6036 | alanine racemase Alr | -0.74 | -1.02 | NS | PA5302 | — | NS | PSPTO0103 | NS |
| PFL_6038 | D-amino acid dehydrogenase, small subunit DadA | NS | -1.10 | NS | PA5304 | — | NS | PSPTO0101 | NS |
| PFL_6074 | putative type VI secretion-associated lipoprotein TagQ | -1.40 | NS | -1.77 | PA0070 | — | NS | — | — |
| PFL_6087 | type VI secretion protein TssB | -1.27 | -0.74 | -2.29 | PA0083 | — | NS | — | — |
| PFL_6088 | type VI secretion protein TssC | -1.09 | NS | -1.81 | PA0084 | — | NS | PSPTO5432 | 0.61 |
| PFL_6089 | type VI secretion system effector Hcp | -1.03 | NS | -2.70 | PA0085 | — | NS | — | — |
| PFL_6128 | aspartate ammonia-lyase AspA | -1.41 | NS | NS | PA5429 | — | NS | PSPTO5499 | 0.84 |
| PFL_6173 | CobW/P47K family protein | NS | -1.75 | NS | PA5535 | — | NS | PSPTO5526 | NS |
| PFL_6175 | hypothetical protein | NS | -1.01 | NS | — | — | — | PSPTO5527 | NS |
| PFL_6177 | GTP cyclohydrolase | NS | -1.46 | NS | PA5539 | — | NS | PSPTO2578 | NS |

^ Pf-5 genes that contain Fur binding motifs upstream.

* Statistically significant difference determined using MultiExperiment Viewer (MeV) software version 4.1 with P-value < 0.01.

NS means not significant when analyzed with SAM in this study as well as the study comparing a *gacA* mutant to the parental strain Pf-5 [35]. Similarly, for the studies on *P. aeruginosa* [27], [28] and on *P. syringae* DC3000 [29], NS means not significant when analyzed according to their respective study criteria.
