# Supplementary material for: The Effect of Iron Limitation on the Transcriptome and Proteome of Pseudomonas fluorescens Pf-5
Source: PLoS One. 2012 Jun 18;7(6):e39139. doi: 10.1371/journal.pone.0039139 (PMC3377617; doi:10.1371/journal.pone.0039139)
Supplement: Table S2 — Primer sequences for qRT-PCR validation of microarray data. (DOC) [file pone.0039139.s006.doc]

Table S2. Primer sequences for qRT-PCR validation of microarray data

| Gene ID | Annotated functions | Primer names | Primer sequences | Product sizes (bp) |
| --- | --- | --- | --- | --- |
| PFL_4858 | bacterioferritin-associated ferredoxin | PFL_4858-qF  PFL_4858-qR | CGACGGACAAATTCGCGAAGCG  CACAGGGTAGGGGATTGCCGC | 172 |
| PFL_4635 | arginine deiminase ArcA | PFL_4635-qF  PFL_4635-qR | AGCGCGAGCAATGGGACGAC  CGCGGATGATCGGGCAGGTC | 189 |
| PFL_4515 | bifunctional N-succinyldiaminopimelate-aminotransferase/acetylornithine transaminase protein | PFL_4515-qF  PFL_4515-qR | GTAGGTGGTGCCGTGGGTGC  AAGTGCAGACCGGCATGGGC | 176 |
| PFL_1649 | flagellar motor switch protein FliN | PFL_1649-qF  PFL_1649-qR | TCGAGCCGCGATGACGATGC  ACGACGAGAAACCGGCGCAA | 159 |
| PFL_4410 | C4-dicarboxylate transporter/malic acid transport protein | PFL_4410-qF  PFL_4410-qR | AACGGCGGGTGATGAGCACG  CGGTGCCTTGGGGATGCTGG | 170 |
| PFL_2095 | translational regulator RsmE | PFL_2095-qF  PFL_2095-qR | AGGGGGTTTCGCGTTTGTCCG  ACCATTCTCGGCGTCAGCGG | 133 |
| PFL_5687 | acyl-CoA dehydrogenase | PFL_5687-qF  PFL_5687-qR | GCCGCCCTGGAAGCCGATTT  TGAACTTCGACGCGGCCACC | 140 |
| PFL_0264 | phosphopantetheine attachment site domain-containing protein | PFL_0264-qF  PFL_0264-qR | CAGGCGGCGGTCGAACTCAA  GTCACCCGCCACCTTCACCG | 156 |
| PFL_2552 | QAT family ABC transporter substrate-binding protein | PFL_2552-qF  PFL_2552-qR | TACGCCAAGGCCCATCCCGA  TGTAGGTGCGGCGGCTGTTG | 166 |
| PFL_2917 | oxidoreductase membrane protein | PFL_2917-qF  PFL_2917-qR | GCGCAGGCGTTGGGTGTAGT  TGCGCGAACCCCATCCGTTC | 101 |
| PFL_2578 | hydrogen cyanide synthase HcnB | PFL_2578-qF  PFL_2578-qR | TTACCTGGGGCCGCGCTACT  TGCTGCACCTGCTCTTCGCC | 156 |
| PFL_5183 | cytochrome c551 peroxidase CcpA_2 | PFL_5183-qF  PFL_5183-qR | CGCGATGCTCATGCCGGAGT  GACCCGCGCCTGTCCAAGTC | 156 |
| PFL_6176 | N-acetylmuramoyl-L-alanine amidase AmiC | PFL_6176-qF  PFL_6176-qR | GCAGCACAGTCCCGCCCAAT  ATGGCCCAGCGGGAAAACGG | 151 |
| PFL_4522 | acetyl-CoA synthetase AcsA_1 | PFL_4522-qF  PFL_4522-qR | CATGAGGGTGGCGCCGGTTT  GTGGGCGAGCCGATCAACCC | 114 |
| PFL_5938 | NAD(P)H-dependent FMN reductase SsuE | PFL_5938-qF  PFL_5938-qR | ACCGGCGTCGCAATCAGCAA  CGGGGTGCTGCTGGAGTTGG | 180 |
| PFL_3806 | 50S ribosomal protein L36 RpmJ | PFL_3806-qF  PFL_3806-qR | GGTTCTTCGCCCCGCCCTG  TCGTCACCGCGACTGCCAGA | 101 |
| PFL_5555 | bacterioferritin Bfr_2 | PFL_5555-qF  PFL_5555-qR | CAGCATCTCCGGCACCGTGG  AACACGCCGACGCCCTGATG | 101 |
| PFL_4190 | extracytoplasmic-function sigma-70 factor PvdS | PFL_4190-qF  PFL_4190-qR | CAGATCACCTCCTCGTTCAA  CGCCATGAATAACCACATTC | 145 |
| PFL_0906 | FagA protein | PFL_0906-qF  PFL_0906-qR | TATTGATCGATACCGCAACC  GCAATTGCACAAAGACAACC | 113 |
| PFL_0909 | superoxide dismutase, Mn  SodA1 | PFL_0909-qF  PFL_0909-qR | AGAAACTGGTGGTGGAAAGC  GACGTTGTAGAAGGCGTTGA | 149 |
| PFL_5965 | 2-octaprenyl-3-methyl-6-methoxy-1,4-benzoquinol hydroxylase | PFL_5965-qF  PFL_5965-qR | CAGACGATCGAACACCAATC  GAGTGGGATTACCTGCATCA | 152 |
| PFL_2291 | RNA polymerase sigma factor, FecI family | PFL_2291-qF  PFL_2291-qR | GCCTGCTGATCGATCTGTTT  GAAAGCCTGTTTCACCTTGG | 176 |
| PFL_5587 | 30S ribosomal protein S12  RpsL | PFL_5587-qF  PFL_5587-qR | CGCCGGAAGTATCCAGGGAGC  TGTGCCGTGTGCGTCTGACC | 165 |
| PFL_5586 | 30S ribosomal protein S7  RpsG | PFL_5586-qF  PFL_5586-qR | ACGCTCGGCAACGGCTTTCT  CGTCGCGTAGCAGCCAAACG | 114 |
