# Supplementary material for: The Effect of Iron Limitation on the Transcriptome and Proteome of Pseudomonas fluorescens Pf-5
Source: PLoS One. 2012 Jun 18;7(6):e39139. doi: 10.1371/journal.pone.0039139 (PMC3377617; doi:10.1371/journal.pone.0039139)
Supplement: Table S3 — Regulation of genes with predicted upstream Fur binding sites. (DOC) [file pone.0039139.s007.doc]

Table S3. Regulation of genes with predicted upstream Fur binding sites. Fold changes are shown in log_2_-based format. – FeCl_2_ and – FeCl_3_ respectively represent FeCl_2_ and FeCl_3_ deprivation studies.

| Gene ID | Putative Fur binding sites* | Annotated functions | – FeCl_2_ | – FeCl_3_ |
| --- | --- | --- | --- | --- |
| PFL_0127 | AATGCGACTAATTATCATT .. 25 | RNA polymerase sigma-70 factor, ECF subfamily | **3.12** | 0.83 |
| PFL_0145 | AATGATAATTGCTCTCAAT .. 19 | RNA polymerase sigma-70 factor, ECF subfamily | **4.40** | NS |
| PFL_0310 | AATGACAAGCATTACCATT .. 37 | TonB-dependent outer membrane receptor | NS | NS |
| PFL_0311 | AATGGTAATGCTTGTCATT .. 206 | conserved hypothetical protein | NS | NS |
| PFL_0984 | AATGCGAACAATTCTTGTT .. 35 | RNA polymerase sigma factor FecI | **1.89** | NS |
| PFL_0985 | TATGAGAATATTTATCATT .. 38 | conserved hypothetical protein | **1.71** | NS |
| PFL_1284 | AACGCCAGGCTTTCTCATT .. 48 | conserved hypothetical protein | NS | **-1.03** |
| PFL_1285 | AATGAGAAAGCCTGGCGTT .. 107 | surface antigen protein | NS | NS |
| PFL_1900 | AATGCCAATATTTATCGTT .. 30 | DNA-binding protein | **1.16** | 0.97 |
| PFL_2291 | AATGCGATTCATTATCAAA .. 17 | RNA polymerase sigma factor, FecI family | **3.27** | NS |
| PFL_2363 | AATGAGAAGCATTATCATT .. 14 | RNA polymerase sigma factor, sigma-70 family | **2.51** | 0.83 |
| PFL_2393 | AATGATAAGAATTCTCATT .. 53 | RNA polymerase sigma-70 factor, ECF subfamily | **1.48** | NS |
| PFL_2490 | GTTGAGAATCTTTTTCATT .. 75 | drug resistance transporter, EmrB/QacA subfamily | **1.06** | 0.81 |
| PFL_2491 | AATGAAAAAGATTCTCAAC .. 61 | transcriptional regulator, TetR family | NS | NS |
| PFL_2529 | TATGATAAGCGTTATCATT .. 31 | RNA polymerase sigma factor PupI | **1.23** | 0.52 |
| PFL_3154 | GAAGACAATCATTCTCATT .. 40 | TonB-dependent outermembrane ferric aerobactin receptor IutA | NS | NS |
| PFL_3313 | ATCGCGATCAATTCCCATT .. 30 | RNA polymerase sigma-70 factor, ECF subfamily | **1.67** | NS |
| PFL_3482 | TATGGTAATGCTTCTCATT .. 122 | transcriptional regulator, MarR family/acetyl transferase, GNAT family | **1.06** | NS |
| PFL_3483 | AATGAGAAGCATTACCATA .. 40 | RNA polymerase sigma-70 factor, ECF subfamily | **1.87** | NS |
| PFL_3612 | AATCATAATTATTCTCATT .. 44 | TonB dependent outer membrane receptor | NS | NS |
| PFL_3623 | CATGAGAAGCATTATCATA .. 51 | ferric enterobactin ABC transporter, periplasmic ferric enterobactin-binding protein FepB | **2.74** | NS |
| PFL_3806 | GTTGATAATAATTCTCATT .. 51 | ribosomal protein L36 RpmJ | **3.20** | **2.67** |
| PFL_4041 | AATGCGAAGCATTACTATT .. 27 | RNA polymerase sigma-70 factor, ECF subfamily | **1.61** | 0.73 |
| PFL_4080 | TATGAGAATTACTATAAAT .. 101 | RNA polymerase sigma-70 factor, ECF subfamily, FpvI | **2.84** | **1.02** |
| PFL_4081 | ATTTATAGTAATTCTCATA .. 209 | efflux transporter, RND family, MFP subunit | NS | NS |
| PFL_4189 | GACGTCATCCGTTCTCATT .. 149 | non-ribosomal peptide synthetase PvdL | **2.59** | NS |
| PFL_4190 | ATTGACAATCATTATCATT .. 82 | RNA polymerase sigma-70 factor, ECF subfamily, PvdS | **5.07** | **3.94** |
| PFL_4625 | GATGACAATCATTATCATC .. 27 | RNA polymerase sigma-70 factor, ECF subfamily | **1.70** | 0.85 |
| PFL_4828 | AATGCAAATCTTTCGCATT .. 61 | insulin-cleaving metalloproteinase outer membrane protein IcmP | **2.12** | NS |
| PFL_4858 | TTTGACAATCATTCTCGTT .. 51 | bacterioferritin-associated ferredoxin, putative | NS | **4.83** |
| PFL_5169 | AATGATATTCATTAGCATT .. 66 | TonB-dependent receptor | NS | NS |
| PFL_5266 | AATGAAAACAATTATCAAA .. 111 | hemin ABC transporter, periplasmic hemin-binding protein PhuT | **1.01** | 0.37 |
| PFL_5332 | AATAAGAATTATTCTCATT .. 26 | hypothetical protein | **4.72** | **4.32** |
| PFL_5378 | ATTGAGATGCATTCGCATA .. 31 | TonB-dependent outermembrane heme receptor HasR | NS | NS |
| PFL_5380 | ATTGAAAATCATTCTCGAC .. 47 | RNA polymerase sigma-70 factor, ECF subfamily, HasI | **1.72** | NS |
| PFL_5381 | GTCGAGAATGATTTTCAAT .. 193 | 3-dehydroquinate dehydratase, type II AroQ_1 | NS | NS |
| PFL_5704 | ATCGCGAATAATTCTCGTT .. 29 | RNA polymerase sigma-70 factor, ECF subfamily | **3.10** | 0.98 |
| PFL_5964 | AATGGGAGTCCTTATCATT .. 32 | ferric iron ABC transporter, FeT family, periplasmic ferric iron-binding protein, putative | **1.78** | NS |

* Sequences indicate putative Fur binding sites found upstream of genes, as identified previously [35], while the numerals after the sequences show the distance of the sites to the predicted start codon of the genes concerned.

NS means not significant when analyzed with SAM at FDR < 1% in this study.

Numbers in bold denote fold changes that surpass the significant differential expression threshold defined in this study, i.e. equal to or exceeding 2-fold change for transcript levels.
