# Supplementary material for: The Effect of Iron Limitation on the Transcriptome and Proteome of Pseudomonas fluorescens Pf-5
Source: PLoS One. 2012 Jun 18;7(6):e39139. doi: 10.1371/journal.pone.0039139 (PMC3377617; doi:10.1371/journal.pone.0039139)
Supplement: Table S4 — Regulation of clusters containing genes encoding sigma factors, TonB-dependent receptors, and anti-sigma factor regulators as determined by SAM analysis. (DOC) [file pone.0039139.s008.doc]

Table S4. Regulation of clusters containing genes encoding sigma factors, TonB-dependent receptors, and anti-sigma factor regulators as determined by SAM analysis. Fold changes are reported in log_2_-based format. – FeCl_2_ and – FeCl_3_ respectively represent FeCl_2_ and FeCl_3_ deprivation studies.

| Gene ID | Putative function^+^ | – FeCl_2_ | – FeCl_3_ |
| --- | --- | --- | --- |
| PFL_0127^ | ECF sigma factor | **3.12** | 0.83 |
| PFL_0126 | anti-sigma factor | **2.27** | 0.93 |
| PFL_0125 | TonB-dependent receptor, hydroxamate siderophore FoxA | NS | NS |
| PFL_0145^ | ECF sigma factor | **4.40** | NS |
| PFL_0146 | anti-sigma factor | **2.88** | NS |
| PFL_0147 | TonB-dependent receptor, hydroxamate siderophore | NS | NS |
| PFL_0984^ | ECF sigma factor Fecl | **1.89** | NS |
| PFL_0983 | anti-sigma factor FecR | **1.71** | NS |
| PFL_0982 | TonB-dependent receptor, citrate FecA | NS | NS |
| PFL_0988 | ECF sigma factor | NS | -0.28 |
| PFL_0989 | anti-sigma factor | NS | NS |
| PFL_0995 | TonB-dependent receptor | 0.57 | NS |
| PFL_1373 | ECF sigma factor | **1.68** | 0.70 |
| PFL_1372 | anti-sigma factor | 0.78 | NS |
| PFL_1371 | TonB-dependent receptor, haem | 0.45 | NS |
| PFL_2291^ | ECF sigma factor | **3.27** | NS |
| PFL_2292 | anti-sigma factor FecR | **1.88** | NS |
| PFL_2293 | TonB-dependent receptor, pyoverdine | 0.82 | NS |
| PFL_2363^ | ECF sigma factor | **2.51** | 0.83 |
| PFL_2364 | anti-sigma factor | **2.50** | NS |
| PFL_2365 | TonB-dependent receptor, haem | 0.55 | NS |
| PFL_2393^ | ECF sigma factor | **1.48** | NS |
| PFL_2392 | anti-sigma factor | 0.79 | NS |
| PFL_2391 | TonB-dependent receptor, pyoverdine | 0.95 | NS |
| PFL_2529^ | ECF sigma factor PupI | **1.23** | 0.52 |
| PFL_2528 | anti-sigma factor PupR | **1.85** | 0.46 |
| PFL_2527 | TonB-dependent receptor, pyoverdine | 0.48 | NS |
| PFL_2746 | ECF sigma factor | NS | NS |
| PFL_2747 | anti-sigma factor | NS | NS |
| PFL_2745 | TonB-dependent siderophore receptor, putative | NS | NS |
| PFL_3156 | ECF sigma factor | **1.05** | NS |
| PFL_3155 | anti-sigma factor | 0.46 | NS |
| PFL_3154 | TonB-dependent receptor, aerobactin IutA | 0.40 | 0.44 |
| PFL_3313^ | ECF sigma factor | **1.67** | NS |
| PFL_3314 | anti-sigma factor | **1.05** | NS |
| PFL_3315 | TonB-dependent receptor, pyoverdine | 0.45 | NS |
| PFL_3483^ | ECF sigma factor | **1.87** | NS |
| PFL_3484 | anti-sigma factor | **1.14** | NS |
| PFL_3485 | TonB-dependent receptor, pyoverdine | NS | NS |
| PFL_3610 | ECF sigma factor | NS | NS |
| PFL_3611 | anti-sigma factor | NS | NS |
| PFL_3612^ | TonB dependent receptor | 0.67 | NS |
| PFL_4041^ | ECF sigma factor | **1.61** | 0.73 |
| PFL_4040 | anti-sigma factor | 0.81 | NS |
| PFL_4039 | TonB-dependent receptor, citrate | 0.76 | NS |
| PFL_4080^ | ECF sigma factor FpvI | **2.84** | **1.02** |
| PFL_2903 | Anti-sigma factor FpvR | 0.65 | NS |
| PFL_4092 | TonB-dependent receptor, pyoverdine FpvA | 0.88 | NS |
| PFL_4625^ | ECF sigma factor | **1.70** | 0.85 |
| PFL_4626 | anti-sigma factor | 0.46 | NS |
| PFL_4627 | TonB-dependent receptor PhuR, haem | NS | NS |
| PFL_5380^ | ECF sigma factor HasI | **1.72** | NS |
| PFL_5379 | anti-sigma factor HasS | **2.74** | NS |
| PFL_5378 | TonB-dependent receptor HasR, haemophore | 0.92 | NS |
| PFL_5704^ | ECF sigma factor | **3.10** | 0.98 |
| PFL_5705 | anti-sigma factor | **3.36** | NS |
| PFL_5706 | TonB-dependent receptor, ferrichrome receptor FiuA | 0.50 | NS |

^ Pf-5 genes that contain Fur binding motifs upstream [35].

^+^ Putative substrates for TonB-dependent receptors [17].

NS means not significant when analyzed with SAM at FDR < 1% in this study.

Numbers in bold denote fold changes that surpass the significant differential expression threshold defined in this study, i.e. equal to or exceeding 2-fold change for transcript levels.
